# A holistic seismotectonic model of Delhi region

**DOI:** 10.1038/s41598-021-93291-9

**Published:** 2021-07-05

**Authors:** Brijesh K. Bansal, Kapil Mohan, Mithila Verma, Anup K. Sutar

**Affiliations:** 1 National Center for Seismology, Ministry of Earth Sciences, Lodhi Road, New Delhi, 110003 India; 2 Geoscience/Seismology Division, Ministry of Earth Sciences, Lodhi Road, New Delhi, 110003 India; 3 Borehole Geophysics Research Laboratory, Ministry of Earth Sciences, Karad, Maharashtra India

**Keywords:** Solid Earth sciences, Seismology, Tectonics

## Abstract

Delhi region in northern India experiences frequent shaking due to both far-field and near-field earthquakes from the Himalayan and local sources, respectively. The recent M3.5 and M3.4 earthquakes of 12th April 2020 and 10th May 2020 respectively in northeast Delhi and M4.4 earthquake of 29th May 2020 near Rohtak (~ 50 km west of Delhi), followed by more than a dozen aftershocks, created panic in this densely populated habitat. The past seismic history and the current activity emphasize the need to revisit the subsurface structural setting and its association with the seismicity of the region. Fault plane solutions are determined using data collected from a dense network in Delhi region. The strain energy released in the last two decades is also estimated to understand the subsurface structural environment. Based on fault plane solutions, together with information obtained from strain energy estimates and the available geophysical and geological studies, it is inferred that the Delhi region is sitting on two contrasting structural environments: reverse faulting in the west and normal faulting in the east, separated by the NE-SW trending Delhi Hardwar Ridge/Mahendragarh-Dehradun Fault (DHR-MDF). The WNW-ESE trending Delhi Sargoda Ridge (DSR), which intersects DHR-MDF in the west, is inferred as a thrust fault. The transfer of stress from the interaction zone of DHR-MDF and DSR to nearby smaller faults could further contribute to the scattered shallow seismicity in Delhi region.

## Introduction

**Figure 1:**
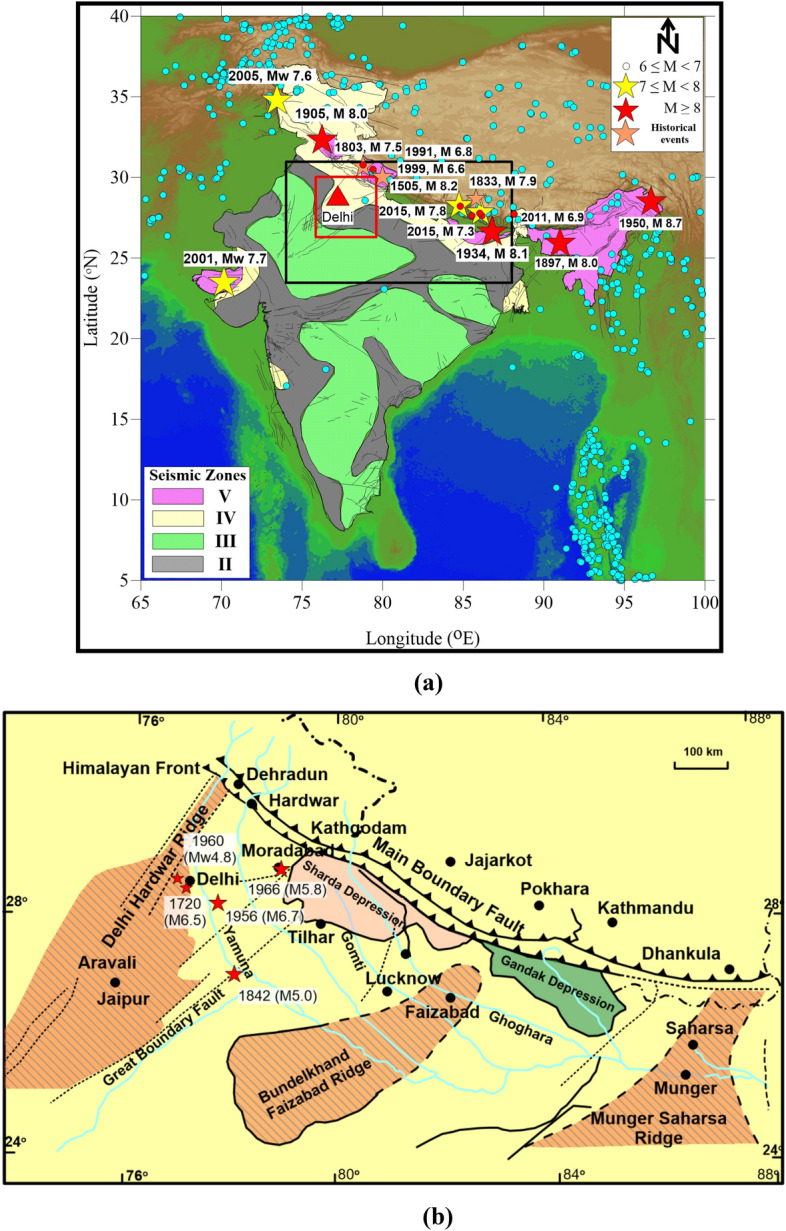
(**a**) Seismicity of Himalaya (magnitude ≥ 4.5 from 01.01.1900 to 10.06.2020) taken from USGS and overlapped on SRTM data of 90 m resolution (http://srtm.csi.cgiar.org). The Ganga basin area is shown with a black rectangle. The red rectangle represents Delhi and surroundings. (**b**) the structure of the basement of the Ganga Basin based on Fuloria1 and Sastri et al.2 overlapped with the epicenter of earthquakes of Delhi region. The figure is prepared using Generic Mapping Tools version 4.4.0^3^.

The National Capital Territory (NCT) of Delhi is located about 250 km away from the seismically active Himalayan collision zone and experiences shaking frequently from far field and near field earthquakes. Delhi is placed in seismic zone IV in the seismic zoning map of India (IS 1893, Part1: 2016) (Fig. 1a). This intraplate region is exposed to moderate to high risk due to Himalayan earthquakes, e.g., Mw 7.5 Garhwal Himalaya in 1803 (1803 GH), Mw 6.8 Uttarkashi earthquake in 1991 (1991 UKS), Mw 6.6 Chamoli earthquake in 1999 (1999 CHM), Mw 7.8 Gorkha earthquake in 2015 (2015 GRK) (Fig. 1a) and a few moderate earthquakes from the Hindukush region as well as local earthquakes, e.g., M 6.5 Delhi earthquake in 1720, M5.0 Mathura earthquake in 1842, M 6.7 Bulandshahar earthquake in 1956 and M5.8 Moradabad earthquake in 1966 (Fig. 1b).

Delhi is one of the largest cities of the country and habitat for ~ 20 million people. It is a socio-economic hub with a wide spectrum of dwellings, from low-income people with poor constructions to very large buildings and infrastructure representing the rapidly growing economy. The seismic activity in Delhi and surroundings has been a cause for concern to the public and also it caused damage to infrastructure from time to time. Recognizing the high-risk potential, a seismic monitoring and hazard evaluation program was initiated for the Delhi region about two decades ago. The continuous monitoring with progressively upgraded network provides new insights into the spatial-depth distribution and source mechanisms.

The existing studies of Delhi Region suggest two contradictory subsurface structural trends: (i) thrust/reverse fault with strike-slip component^4^ and (ii) normal fault with strike-slip component^5,6^ (Supplementary Table 1)**.** However, in absence or with limited subsurface geophysical information, the focal mechanisms and depth distribution of earthquakes prove to be helpful to guide identification of seismogenic structures/faults.

Recently, three earthquakes occurred in Delhi region (12 April 2020 of M3.5, 10 May 2020 of M3.4 and 29th May 2020 of M4.4), which have been recorded by a dense local network of 15 seismic stations. Taking advantage of the availability of good quality of recorded data, the faulting mechanisms of these moderate events along with two past events (01st June 2017 of M 4.2 and 29th May 2011 of M3.4) are determined for re-examination of structural trends. A comprehensive appraisal (seismological and geophysical) has also been conducted to probe linkages within local geological structures of the region and to propose a holistic seismotectonic model.

## Geology and tectonic setting of Delhi region

The Ganga basin, with an area of about 250,000 sq km falls within Long. 77° E–88° E and Lat. 24° N–30° N (Fig. 1b). It is located between the northern fringe of the Indian peninsula and the Himalaya and extends from Delhi-Hardwar Ridge (DHR) in the west to Munger-Saharsa ridge in the east (Fig. 1a,b). Delhi is located near the northern fringe of the Proterozoic Aravalli-Delhi fold belt and western edge of the Ganga basin (Fig. 1b).

The terrain is generally flat except for a low NNE-SSW trending Delhi Hardwar ridge in the southern and central part of the area which consists of Quartzite while the Quaternary sediments, comprising the older and newer alluvium, cover the rest of the area. The thickness of the alluvium, both on the eastern and western side of the ridge, is variable but west of the ridge it is generally thicker (290 m).

The thick deposits of soft sediments of Yamuna plains plays a dominant role in ground motion amplification as experienced during past earthquakes^7^.

Historically, studies of the Himalayan foot-hills belt were initially conducted by Medlicott^8^, Theobald^9^, Oldham^10^, and Middlemiss^11^. Later, Wadia^12^ and Auden^13^ and several other officers of the Geological Survey of India mapped different parts of this belt. Agocs^14^ provided the first geophysical (aeromagnetic) data for the sedimentary thickness and configuration of the basement in the Indo-Gangetic plains. Krishnan and Swaminath^15^ proposed that the great Vindhyan basin must be extended into the Lesser Himalayan region. Sengupta^16^ using the aeromagnetic data subdivided the Ganga basin into four parts separated by basement ridges or faults or both (Fig. 1b). Based on a re-interpretation of earlier gravity data, Sengupta^17^ (1964) correlated the evolution of the Himalaya with the subcrustal movements below the Gangetic plains. The Oil and Natural Gas Commission, based on geophysical surveys (aeromagnetic, gravity, and seismic) and drilling data, in 1968 identified the three ridges in the Ganga basin named (from east to west) as Munger-Saharsa ridge, Faizabad ridge and Delhi-Hardwar Ridge (DHR) (Fig. 1b). Valdiya^18^ correlated the transverse structures in the Himalaya to these three hidden basement ridges. The western boundary of the Ganga basin is delineated by DHR and the eastern margin by the northeastward continuation of the buried basement ridge (Munger-Saharsa ridge)^2^. The DHR was proposed with the least areal extent (6000 sq km) among all three ridges. Sastri et al.^2^ and Karunakaran and Ranga Rao^19^ described the shallow character of the DHR. The ridge was not traced with seismic survey beyond Meerut; ‘the trend is probably obscured by a thick Neogene cover’^6^.

**Figure 2:**
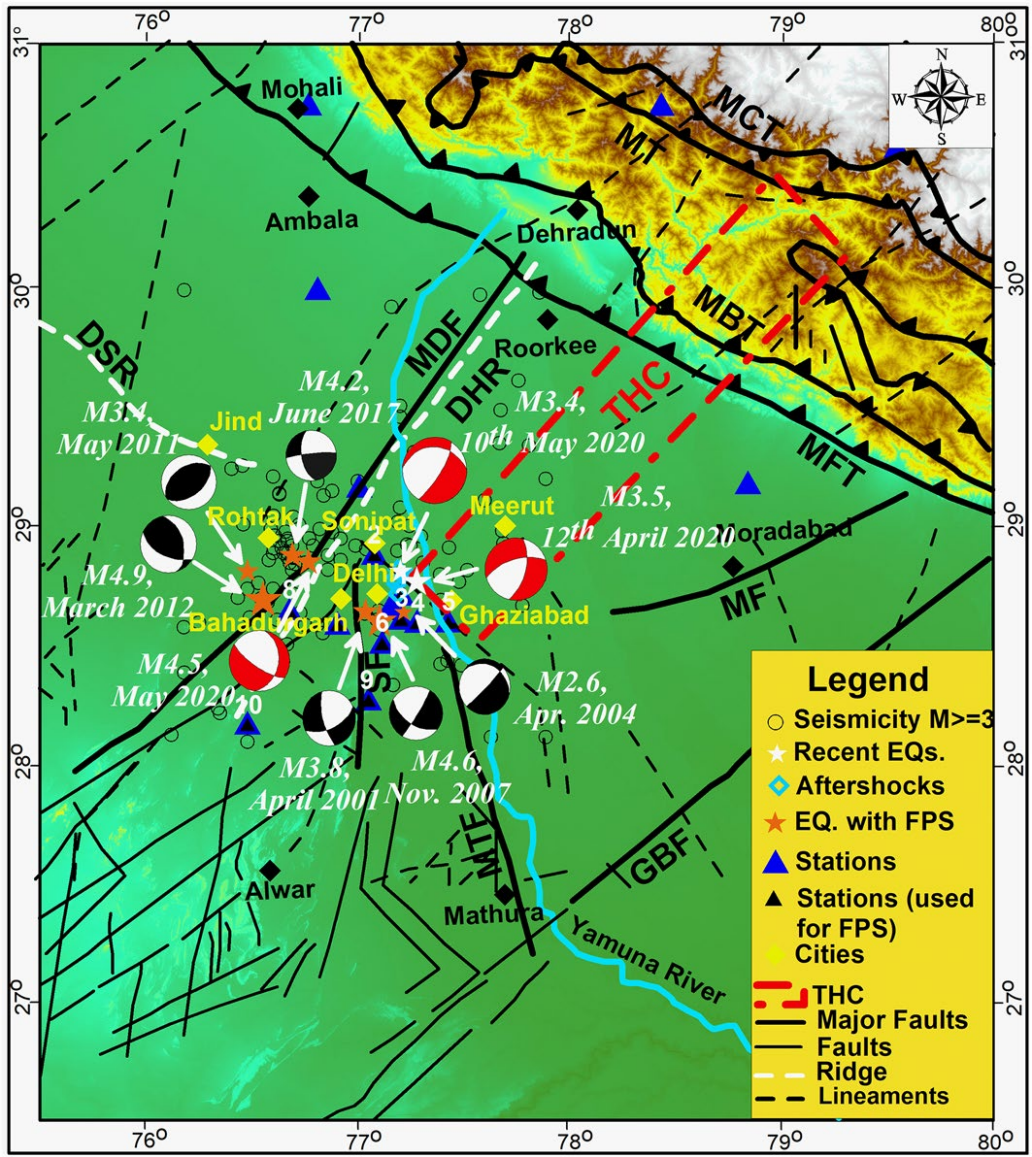
Tectonic map of the Delhi region with the Trans- Himalayan Conductor (THC) superimposed on it. The earthquakes with magnitude M > 3.0 from 2001 to 10th June 2020 are plotted (stars) from the earthquake catalog prepared by NCS, New Delhi. Major tectonic features of the Himalaya; Main Boundary Thrust (MBT), Main Central Thrust (MCT) and Main Frontal Thrust (MFT) are shown along with the regional tectonic features including Mahendragarh–Dehradun Fault (MDF), Delhi–Hardwar Ridge (DHR), Moradabad Fault (MF), Sohna Fault (SF), Mathura Fault (MTF) and Great Boundary Fault (GBF). The fault plane solutions of the four past earthquakes are shown with black & white beach balls prepared from and the recent (12th April 2020, 10th May 2020 and 29th May 2020) earthquakes are shown with red & white beach balls. The stations used for computation of fault plane solutions are shown with black triangles and station numbers (1:NDI; 2:NRLA; 3: LDR; 4:JMIU; 5:BISR; 6:AYAN; 7:UJWA; 8:JHJR; 9:SONA; 10:KUDL). The tectonic features are from the files provided at BHUKOSH portal of Geological Survey of India (http://bhukosh.gsi.gov.in/Bhukosh/MapViewer.aspx). These features are overlapped on SRTM data of 90 m resolution (http://srtm.csi.cgiar.org). The figure is prepared using Generic Mapping Tools version 4.4.0^3^.

Based on magnetic survey, Arora et al.^20^ proposed a major conductive structure, namely, the Trans Himalayan conductor (THC), that strikes perpendicular to the Ganga basin into the foothills of the Himalaya and located east of Delhi (Fig. 2). Later, through a magnetic survey, Arora and Mahashabde^21^ characterized the THC as a major electrical conductive structure (having a resistivity of 2 Ohm.m) with a width of 45 km and depth of 15 km following the strike of the Aravalli range and running into the Himalaya (Fig. 2).

Mallick et al.^22^, following the study of Raiverman et al.^23^, have suggested a deep-seated fault along the course of the Yamuna River formed by the flexure of the Indian Plate due to subduction beneath the Himalaya. Valdiya^24^ and Chandra^25^ have also indicated a fault zone along the strike of Aravallis in this area. By correlating seismicity with the changes in the Coulomb stress, Arora et al.^26^ proposed along-strike segmentation of NW Himalaya, controlled by the subsurface ridges (underthrusting the Indian Plate) and by rift and nappe structures. They suggested the episodic reactivation of Delhi-Hardwar Ridge due to the strains resulting from the locking of Indian-Eurasian Plates as proposed by Arora^27^.

Dubey et al.^28^ inferred three NW–SE trending reverse faults in the Delhi region using Remote Sensing, Ground-Penetrating Radar (GPR), and Bouguer gravity anomaly data. However, due to very limited depth of penetration of GPR survey (a few meters), modern geophysical surveys with a higher depth of penetration (e.g., Magnetotellurics / Seismic) are imminent to verify and precise characterization of these faults. Dubey et al.^28^ have also suggested that earthquakes that occurred near Rohtak and have orientation other than MDF (i.e. NE-SW) might be related to lithospheric crustal loading of the Himalaya orogeny on the Delhi-Sargoda Ridge. Based on the gravity and aeromagnetic investigations, GSI^29^ proposed a NE-SW trending, 295 km long fault linking Indian peninsular craton in the south to Himalayan Frontal Thrust (HFT) in the north along the DHR and named it as MDF. At the junction of MDF and HFT, Jade^30^ estimated the convergence rate of 10–18 mm/year between India and Tibet. The information on slip rates along major faults of Delhi region is not available. Patel et al.^31^ delineated the shallow steep vertical faults near MDF (though MDF was not traced) using GPR survey and suggested MDF as a normal fault system at shallow subsurface and showing normal with oblique-slip motion.

**Figure 3:**
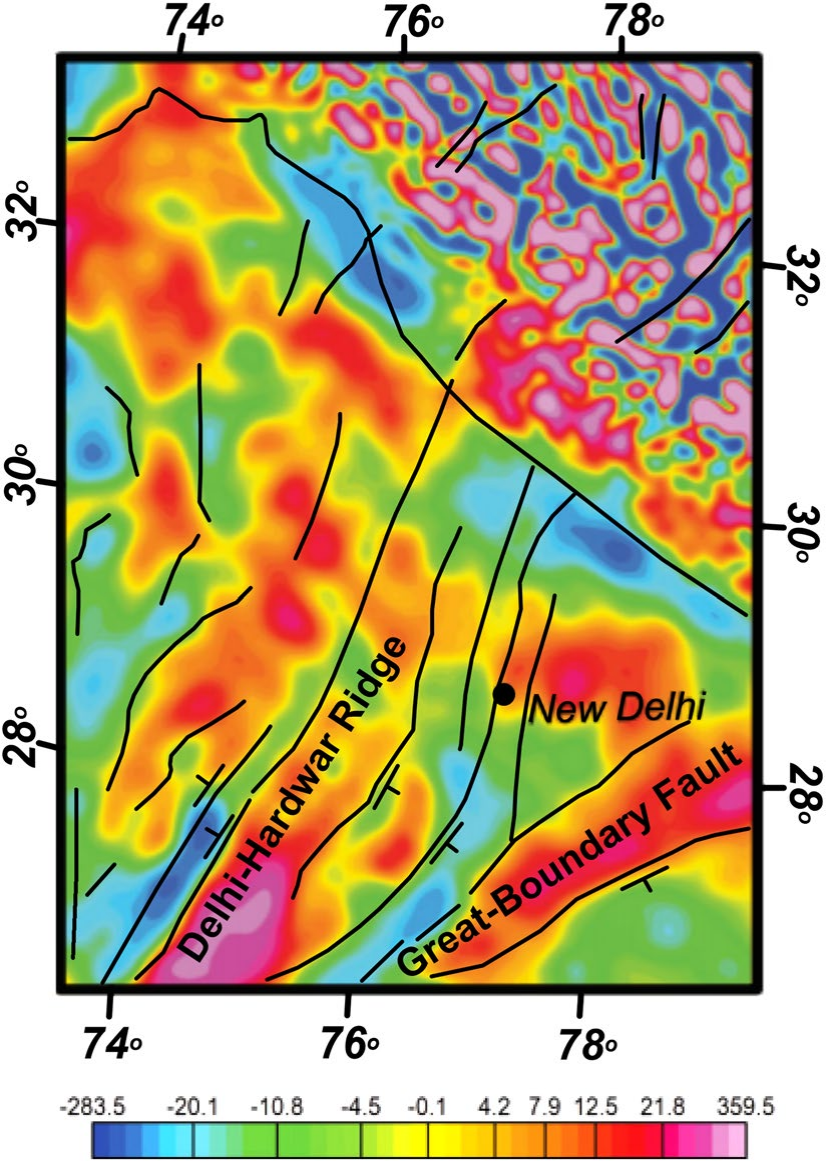
Bouguer gravity map (in Gal) of Delhi region with lineament interpretation and dip directions (modified after Goddin and Harris^39^ Harris^33^).

Ravi Kumar et al.^32^ published the Bouguer anomaly map of North India including the Ganga basin using well-controlled ground data and inferred that the Aravalli Delhi Mobile Belt (ADMB) and its marginal faults extend to the Western Himalayan front via Delhi where it interacts with the Delhi–Lahore ridge and further north with the Himalayan front causing seismic activity. Godin and Harris^33^, using Bouger gravity data of Delhi region derived from Earth Gravitational Model (EGM) 2008 have suggested that NE Delhi–Hardwar trend continues northeastward across the surface trace of the Main Frontal Thrust to the Karakoram fault. They further suggested that the DHR is delimited by the Shimla and Dehradun lineaments and proposed it as a horst with steeply-dipping normal faults on either side (Fig. 3). The Dehradun lineament connects the eastern edge of the Delhi–Hardwar Ridge to the Burang graben north of Shimla, the westernmost N–S graben of southern Tibet.

Dwivedi et al.^34^, through 3D structural inversion of gravity data (from Gravity Map (WGM)-2012 and gravity map series of India-2006 (GSI-NGRI, 2006)), speculated that NE trending Delhi Fold Belt deflected westward towards the shallower DSR and produce clustered seismicity in the hinge zone of this crustal bending near the Delhi region. They suggested that it is happening due to development of high strain resulting from crustal buckling of Delhi Fold Belt and DSR. They opined that the structural setup possibly developed after NW corner indentation and anti-clockwise rotation of Indian plate (post-Eocene collision) (as proposed by Voo et al.^35^) led to the westward deflection of NE trending Delhi Fold Belt. In addition to geophysical and geological studies, the seismological studies have a special contribution in understanding the subsurface structures and seismotectonics of the region.

## Seismic monitoring in Delhi region

Among the far-field moderate to large earthquakes (1803 GK, 1991 UKS, 1999 CHM, 2015 GRK) experienced in the Delhi region from Himalayan sources, the earthquake of 1st September 1803 (1803 GH) is considered to be important as damage was observed in Delhi and its surrounding region. Different locations were proposed for this earthquake. Initially, this earthquake was considered as the 1803 Mathura earthquake (M 6.8)^10,36,37^. Later, it was studied in detail^38,39^ and suggested renaming the event as the 1803 Garhwal earthquake.

As mentioned in the preceding section, the Delhi region has also experienced near-field earthquakes from the local sources [Historical earthquake of Delhi (M6.5, 1720); Bulandshahar earthquake (M 6.7, 1956); and Gurgaon earthquake (M 4.8, 1960)] (locations given in Fig. 1b and Supplementary Table 2). The intensity of the 1720 Delhi earthquake was assessed as IX in the Old Delhi area. Though the exact epicenter of this event is uncertain; it was in the vicinity of Delhi^40^. The 1956 Bulandshahar earthquake was felt over a larger area and deaths as well as destruction to property were reported. The 1960 Gurgaon earthquake (M4.8) is the closest instrumentally located event to the Delhi region, though the location and magnitude were debated (Supplementary Table 2).

Seismic instrumentation in the Delhi region started in 1960 by India Meteorological Department (IMD) and initially, an analog seismological observatory was installed at Delhi Ridge. This observatory was later upgraded to the World-Wide Standardized Seismograph Network (WWSSN) standard in 1963^41^. The seismograph installed at the observatory recorded a large number of microtremors including, those originated from Sonipat area (about 50 km NW of Delhi) (Fig. 2) during the swarm activity of the Sonipat-Rohtak area (NW of Delhi) in 1963–65. The seismicity during swarm activity of 1963–65 was found to be concentrated in three clusters, namely, west of Delhi, near Sonipat, and close to Rohtak^42,43^. An analog observatory was established at Lodi Road area in the southern part of Delhi (Fig. 2, given with label no. 3 having code LDR) in 1964. Later, in 1974, analog seismological observatories were installed at three other locations, Rohtak, Sohna and Meerut adjoining Delhi. On 28 July 1994, an event of magnitude M4.0 was recorded in Delhi and reported to have caused damage to one of the minarets of Jama Masjid^44^. In year 2000–2001, 16 stations (12 stations with single component and four stations with three-component seismographs) VSAT based Digital Seismic Telemetry Network was established for close monitoring of earthquake activity in Delhi region. Nine field stations in Digital Seismic Telemetry Network were deployed within a radius of 80 km of Delhi. The two earthquakes of magnitudes M4.0 and M3.8 that occurred on 28.02.2001 and 28.04.2001, respectively, in Delhi region were recorded by the DTSN.

The second swarm activity in the Jind area (~ 80 km NW of Sonipat and ~ 130 km NW to Delhi (Fig. 2) occurred during the period December 2003–January 2004 and observed in two clusters. This swarm activity was characterized by 152 tremors, out of which 62 events were of magnitude (ML) range 0.5–3.4^45^. Shukla et al.^46^ correlated the seismicity clusters with the NW–SE trending Delhi Sargoda ridge (DSR).

All the 16 stations of Delhi Seismic Telemetry Network were upgraded, and 9 new stations were installed during 2015–2018 in and around Delhi. These stations are equipped with VSAT for receiving data in real time at the National Center for Seismology (NCS), New Delhi. Presently these stations are integrated with the National Seismological Network, which is now a state-of- the-art network with 115 broadband, three-component seismographs spread across the entire country and has real-time data reception from field stations to Central Receiving Station (CRS) in New Delhi. The data are analyzed in CRS and the information is disseminated for follow-up actions.

From the analysis of past data, it is observed that 122 earthquakes of magnitude M ≥ 3.0 including, eight earthquakes with magnitude M ≥ 4 (with the largest earthquake of M4.9 on 05th March 2012) occurred in Delhi region during January-2001 to 10thJune 2020 (Supplementary Fig. 1a). The depth distribution of the events is shown in Supplementary Fig. 1b. Focal depths generally lie within 15 km from the surface (with a depth uncertainty of ~ 2–4 km) with only about 10% events being deeper than 15 km (Supplementary Fig. 1b). In recent years, M4.9 March 2012, M4.6 September 2016, and M4.6, June 2017 earthquakes are the significant local earthquakes recorded in the Delhi region.

Richter^47^, studied the seismotectonics of the Delhi region and suggested that the region east of Delhi may be associated with the block faulting. Chouhan^42^ studied the seismicity of Delhi using 74 earthquakes of magnitude ≥ 2.0 (for a period between 1962 to 1972) and suggested that most of the seismically active areas lie at the junction of Delhi-Hardwar Ridge, the Lahore-Delhi ridge (DSR) and the axis of Delhi Fold Belt. Molnar et al.^4^ studied the 10th October 1956 Bulandshahr earthquake (located east of DHR) and suggested the fault plane solution as normal faulting focal mechanism. Chouhan^42^ has also estimated the fault plane solution of October 10, 1956, and August 15, 1966, earthquakes occurred near Bulandsahar and Moradabad, respectively (both falls in the east to DHR) and suggested a steep dip, strike-slip with small normal component faulting.

Shukla et al.^46^ used first-motion data recorded by the Delhi Telemetry Seismic Network (between 2001–2004) to determine the focal mechanism of small 19 local earthquakes and suggested the thrust with the strike-slip focal mechanism. They also associated seven earthquakes with MDF and proposed a reverse fault mechanism on a steeply dipping plane (Dip 60^o^to 85°). They further proposed MDF as a strike-slip fault and reactivated as “thrust” with strike-slip component ‘in the imparted tectonic domain of back thrust’. The statement seems contradictory as the fault plane solution estimated by them suggested a steep dip (of 64^o^ to 85°).

Bansal et al.^5^ estimated the source characteristics (including depth and focal mechanism) of the two earthquakes (28th April 2001 and 18th March 2004) in Delhi and provided valuable new information. The focal mechanism of the earthquakes have shown normal faulting with a large strike-slip component (having Dip of 64^o^ to 85°) (Fig. 2) with one of the nodal planes in NE–SW direction. Singh et al.^6^ also analyzed the 25th November 2007 (Mw 4.1) earthquake in detail and given the strike-slip faulting with some normal component mechanism (having dip of 55° to 86°) (Fig. 2). Shukla et al.^46^ used 6 to 10 first motions for estimating the focal mechanism of small-magnitude earthquakes which are insufficient/ and are often difficult to read and focal mechanisms may not be well-constrained^5^. Though Singh et al.^6^ emphasized that the focal mechanism estimated by Bansal et al.^5^ through well recorded data from Digital Strong Motion Network of Central Building Research Institute, Roorkee is reliable.

## Fault plane solutions and structural trends

Recently, three earthquakes occurred on 12th April, 10th May and 29th May 2020 were recorded by the more than 22 stations of the National Seismological Network (NSN), distributed in the northern part of India. The fault plane solution (FPS) of the event of 12thApril 2020 event has been estimated by Pandey et al.^48^. The NSN has also reported two more earthquakes of M>3.0 on 29th May 2011 and 01st June 2017. The fault plane solutions of these four events (29th May 2011, 01st June 2017, 10th May 2020 and 29th May 2020) are determined in the present work using the ISOLA software package^49^ and given in Table 1 along with the FPS of 12th April 2020 determined by Pandey et al.^48^. Only those stations with cut-off signal to noise ratio >2 in the frequency range of interest are used for estimation of fault plane solutions of these events (Fig. 2). The computational details are given in Supplementary data.

**Table1:** Source parameters of recent earthquakes of 12th April, 10th May 2020 and 29th May 2020, 01st June 2017 and 29th May 2011 in the Delhi region.

Origin date and time (dd/mm/yy & hh:mm:ss)	Lat (^o^N)	Long (^o^E)	Focal Depth in km	Str1	Dip1	Rak1	Str2	Dip2	Rak2	Mw
29/05/2011 00:05:39.0	28.80	76.49	10	236◦	41◦	94◦	52◦	50◦	87◦	3.4
01/06/2017 22:55:57.0	28.85	76.70	13	264◦	83◦	31◦	171◦	59◦	172◦	4.2
12/04/2020 12:15:02.3	28.752	77.265	16	13◦	55◦	− 135◦	253◦	55◦	− 45◦	3.5
10/05/2020 08:15:29.17	28.797	77.283	14	32◦	75◦	− 117◦	275◦	31◦	− 31◦	3.4
29/05/2020 15:38:40.35	28.838	76.764	13.9	10◦	37◦	− 29◦	124◦	73◦	− 123◦	4.5

The FPS of 12th April 2020 shows two nodal planes striking at 13° and 253° with a dip of 55° each. The two nodal planes show rake of -135 (normal right lateral oblique) and -45 (normal left-lateral oblique) with dominant normal fault mechanism (Table 1). The FPS of 10th May 2020 shows two nodal planes striking 32° and 275° with a dip of 75° and 31^o^ each. The two nodal planes show rake of -117 (normal right lateral oblique) and -31 (normal left-lateral oblique) with dominant normal fault mechanism (Table 1). The second nodal planes of both these earthquakes have suggested the strike of 253° and 275° (Table 1), respectively, which are not consistent with either the trend of Aravalli belt (NNE-SSW) or with the trend of major fault lines (NNE-SSW to N-S) in the region (Fig. 2). Therefore, the nodal planes with strikes of 13° and 32° that are consistent with the Aravalli and major tectonic trends and are considered.

The FPS of 29th May 2020 shows two nodal planes striking at 10° and 124° with a dip of 37° and 73^o^ each. The two nodal planes show rake of -123 (normal right lateral oblique) and -29 (normal left-lateral oblique) with dominant normal fault mechanism (Table 1). Both of the fault plane solutions have suggested a causative fault trending NNE-SSW direction with a steep dip to the NE. The earthquake of 29th May 2020 which occurred ~ 34 km west of the India Gate area of New Delhi (or ~ 18 km east of Rohtak) falls close to MDF.

The FPS of 29th May 2011 shows two nodal planes striking at 236° and 52° with a dip of 41^o^ and 50^o^ each. The two nodal planes show rake of 94 (reverse) and 87 (reverse) with dominant reverse fault mechanism (Table 1). The FPS of 01st June 2017 shows two nodal planes striking at 264° and 171° with a dip of 83^o^ and 59^o^ each. The two nodal planes show rake of 31 (reverse left-lateral oblique) and 172 (right-lateral strike-slip) with dominant reverse fault mechanism (Table 1). Both these earthquakes (29th May 2011 and 01st June 2017) were located close to DSR (~18 km and ~07 km west of MDF, respectively).

An earthquake of magnitude M4.9 was occurred on 5th March 2012 about 20 km south-west of this earthquake. Bansal and Verma^50^ proposed a strike of N348^o^ and a dip of 48° with a rake of 131° (strike slip motion with reverse component) for this earthquake. The earthquake was located ~ 4 km west of the MDF. The estimated fault plane solution has suggested a NNW-SSE trending reverse fault; therefore, it may not be strictly associated with the MDF. Therefore, it is inferred that the earthquakes occurring to the east of DHR/MDF are following the normal with strike slip mechanism and the earthquakes located to the west of DHR/MDF follows the reverse with strike-slip mechanism.

Further, the earthquakes of 12th April 2020 and 10th May 2020 are located to the east of the DHR and south-western edge of the Trans Himalayan Conductor (THC) proposed by Arora et al.^19^ (Fig. 2). FPS of both the recent earthquakes have shown strike of NNE and steep dip of 55°-75° that are commensurate with the geometry of the edge of THC (Fig. 2). The depth of ~ 15 km has been estimated for both these events. Therefore, both the recent earthquakes (12th April 2020 and 10th May 2020) might be nucleated at the southwestern edge of the THC.

## Distribution of strain energy in Delhi region

The energy is a direct indicator of the size of an earthquake and estimation of the accumulated energy in a region can provide valuable information regarding the potential seismic hazard of the region. The spatial variation of energy release can provide information on the potential locales of stress accumulation in a region in the absence of geodetic data. Similarly, the temporal variation of seismic energy of a region can provide the different stages of energy release process^42,51^ and could be used as a long-term earthquake precursor. In the present study, the spatial distribution of strain energy has been estimated for Delhi and the surrounding areas based on the earthquake catalog of the region for the period 1998-2020 taken from the website of National Centre for Seismology (https://seismo.gov.in/content/seismological-data). Earthquakes with magnitude range between M0.8 and M5.1 have been considered. The computational details are given in Supplementary material.

**Figure 4:**
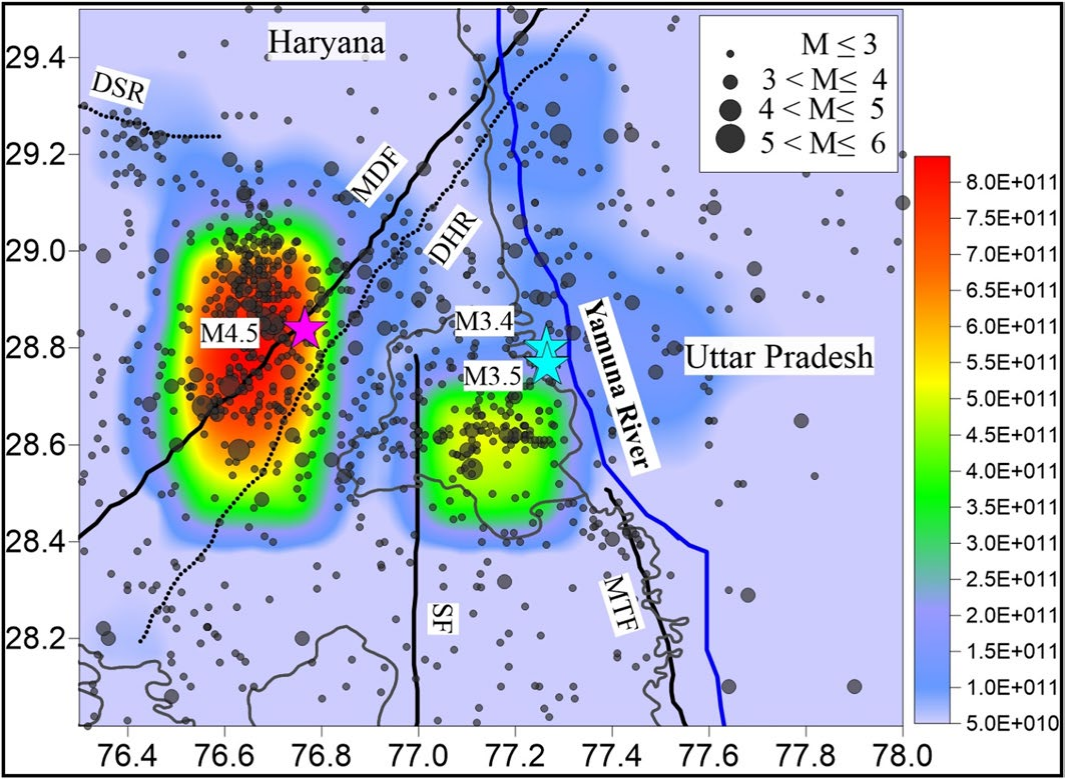
Distribution of estimated seismic energy release from earthquakes during 1998-April 2020 in the Delhi region considering a 0.3° × 0.3° grid. The tectonic features are from the files provided at BHUKOSH portal of the Geological Survey of India (http://bhukosh.gsi.gov.in/Bhukosh/MapViewer.aspx).

The spatial distribution of strain energy estimated in the Delhi region has been shown in Fig. 4. The maximum energy, in the range of 08*10^11^ Joule has been released in this period in the Delhi region. The energy is released mainly in two areas, (i) the area west of DHR/MDF, and (ii) the area east of the DHR (central and NW Delhi). Almost twice the amount of energy has been released in the western part (at the contact zone of DHR and DSR), compared to the eastern part (east of DHR).

During an earthquake, the normal faulting is caused by the gravitational potential, however in case of reverse and strike slip fault, the energy is accumulated as elastic potential. The rocks get deformed under compression, are characterized by yield stresses about 10 times larger than yield stresses in tensional stress fields^52^. Additionally, reverse faults need more energy to move the rocks as compared to thrust in case of reverse fault, the hanging wall moves against gravity. Therefore, the energy dissipation in reverse fault is always more than the thrust and normal faulting. In Delhi region we infer the reverse faulting in the western part of DHR-MDF and normal faulting in the eastern part.

## Proposed seismotectonic model

**Figure 5:**
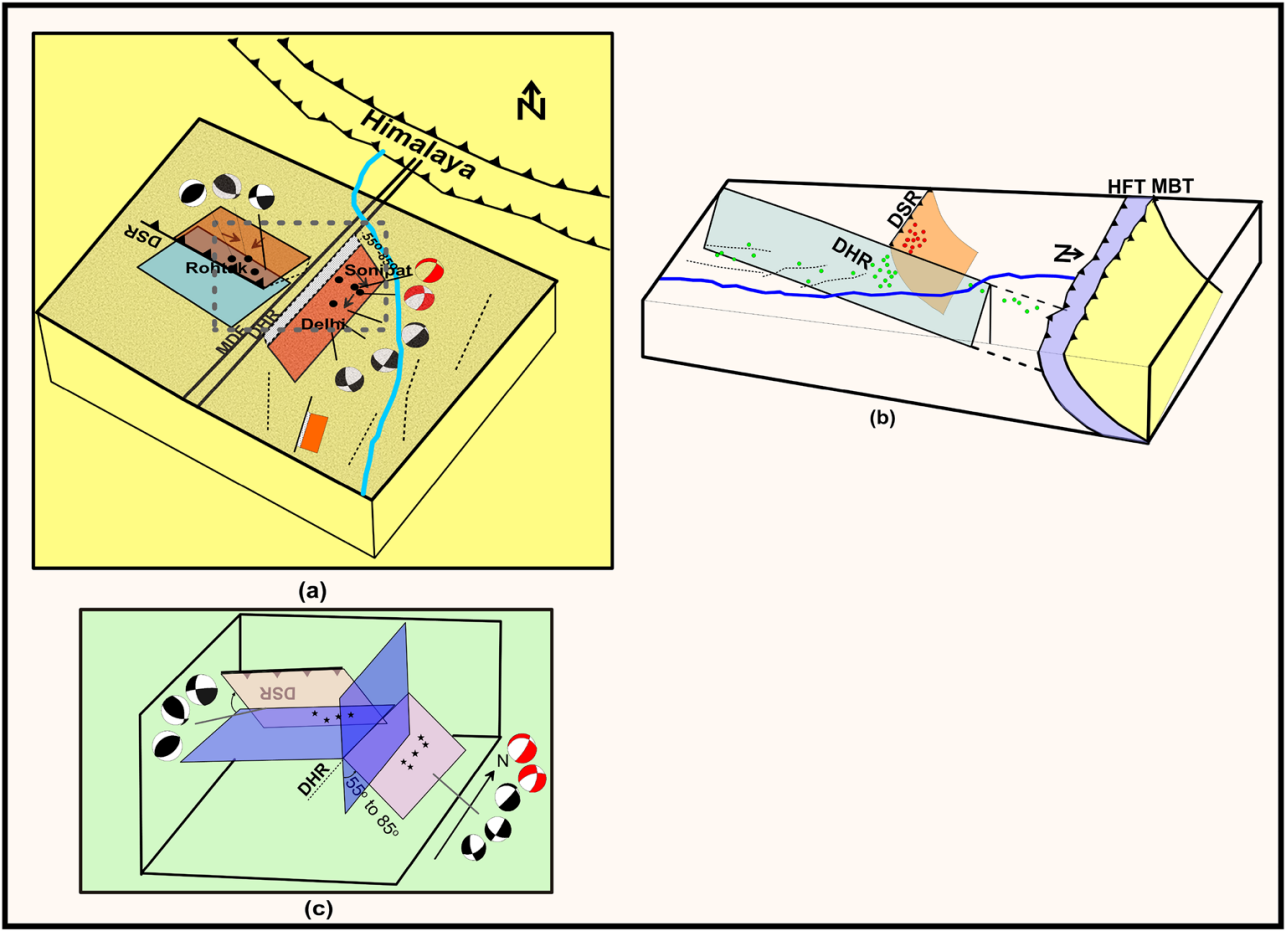
(**a**) Proposed earthquake mechanism model (unscaled)of the Delhi region, (**b**) planer close view of the earthquake process and (**c**) east facing view of the earthquake process of the Delhi region. *DSR* Delhi Sargoda Ridge, *MDF* Mahendragarh-Dehradun Fault, and *DHR* Delhi Hardwar Ridge. The black and white beach balls correspond to past earthquakes and red & white beach balls show the mechanisms of the recent earthquakes of 12th April 2020 and 10th May 2020.

In general understanding, a seismotectonic model suggests the correlation of seismicity with the fault lines of the area and the slip rates along these faults. Delhi area is, however, under-represented in geodetic studies as the GPS network is to be established and no study on Active fault mapping is available along the major faults to confirm slip/slip rates. Taking advantage of the quality data generated by the local seismological network, we propose a seismotectonic model of the Delhi region through the integration of seismicity characteristics, the associated structural features and the estimates of strain energy released in the region. From subsurface structural appraisal, it is inferred that the MDF/DHR follows the trend of Aravalli Delhi Mobile Belt and is a NE-SW trending horst structure with steep normal faulting that continues northeastward across the surface trace of the Main Frontal Thrust to the Karakoram fault. The past seismological studies^4–6,5^ have also suggested normal with a strike-slip focal mechanism in the area east of DHR/ MDF. The focal mechanisms of three recent earthquakes (M 3.5, 12th April 2020 and M3.4, 10th May 2020 and M 4.4, 29th May 2020) to the east of DHR/MDF have also shown normal with strike-slip focal mechanism. The energy released in the last two decades also indicates a similar focal mechanism. Shukla^46^ through FPS of 10 earthquakes (of 2001–2004, with single component seismographs) and Bansal and Verma^51^ through FPS of 01 earthquake recorded with digital strong motion data have suggested DSR, bounded by reverse fault/thrust is an NW–SE trending structure. The fault plane solutions of the two earthquakes (29th May 2011 and 01st June 2017) fallen close to DSR have also shown reverse/thrust faulting with strike slip mechanism. The DSR appears to interact with the DHR in the west of Delhi (Fig. 5a,c). The interaction zone of DHR/MDF and DSR is seismically most active in last two decades (Fig. 2). The scattered and mostly shallow focus seismic activity in the region is inferred to be associated with the presence of faults that got activated time and again due to transfer of stress from the interaction zone of DHR-MDF and DSR. The seismotectonic constraints are presented in the form of a model in Fig. 5.

We have also examined the possibility of THC as the causative structure of two recent earthquakes, M3.5, 12th April 2020 and M3.4, 10th May 2020. Due to high conductivity, the THC might be filled with mantle derived fluid and is ductile in nature. Hence it may not allow large accumulation of strain. Therefore, the strain can be accumulated on both lateral edges of the THC (Fig. 2). Since the FPS of two recent earthquakes (of M3.5, 12th April 2020 and M3.4, 10th May 2020) has shown strike of 13–32°, i.e., NNE, and steep dip of 55°-75^o^to the NE, we reject the possibility of THC as a causative structure in those cases.

## Discussion and conclusions

The densely populated, socio-economically important, housing national capital, the Delhi region has been experiencing earthquakes from both regional (Himalayan) and local sources. The seismicity due to local earthquakes, although not associated with significant damage to property, has created panic among the public in the epicentral areas. The estimation of focal mechanism of recently occurred earthquakes of 12th April 2020 (M3.5) and 10th May 2020, (M3.4) and 29th May 2020 (M4.4), the spatial distribution of energy in the Delhi region in last two decades and appraisal of local sources (geological, geophysical, and seismological) have been integrated to understand the causative structural sources and mechanism of seismicity in the Delhi region. The fault plane solutions of these three earthquakes have suggested a nodal plane trending NNE-SSW direction with a steep dip of 37° to 75° in the NE direction and normal with strike-slip mechanism.

Our analysis on appraisal of subsurface structures of the region suggests three probable major earthquake mechanisms in the Delhi region: (i) episodic reactivation of DHR due to the strains resulting from the locking of Indian-Eurasian plate, (ii) lithospheric crustal loading of the Himalayan orogen on the Delhi-Sargoda Ridge, (iii) interaction of Aravalli Delhi Mobile Belt with Delhi–Lahore Ridge and further north with the Himalayan front.

Since the year 2000, low level seismicity has been observed in the northern part of the Delhi within the zone of interaction of DHR and Himalayan Frontal Thrust. Therefore, the seismic potential of minor to moderate earthquakes cannot be ruled out due to transfer of stress from the intersection zone (of DHR and Himalayan Frontal Thrust) through DHR. A simplified subsurface mechanism of seismicity in the Delhi region has also been proposed in the current study. We believe that the proposed seismotectonic model will serve as a starting model for future studies. NCS has already planned to enhance the local seismic network in the region to help in precise location of smaller events and constraining their focal depths accurately. Also, other investigations such as active fault mapping and subsurface imaging, which are in pipeline would help in refining and strengthening the proposed model.

The seismological investigations, energy estimation and appraisal of subsurface structures have led to the following major results: (i) Two major ridges (DHR and DSR) are interacting in the west of Delhi NCT; DSR is bounded by thrust/ reverse faulting and DHR is a steep horst structure associated with normal faulting, (ii) The earthquake in the Delhi region have occurred in two major clusters in last two decades; each is located west and east to DHR/ MDF, (iii) Higher seismic energy (almost two times) has been released in the western cluster located west to Delhi as compared to eastern cluster, (iv) two major structural/ fault mechanisms in the region have been recognized-thrust/ reverse with strike slip mechanism in the western part and normal with strike slip mechanism in the eastern part due to the presence of DHR/ MDF associated with steep normal faulting in the east and presence of DSR bounded by thrust in the west, respectively, and (v) The scattered shallow seismicity in the region is inferred to be due to stress transfer from interaction zone of two ridges (DHR and DSR) to the nearby faults.

## Methods

The earthquakes occurred on 10th May (M3.4), 29th May 2020 (M 4.4), 29th May 2011 and 01st June 2017 are recorded up to 32 broad band seismometers. The events were located using the Seisan software package^53^ maintaining a small root mean square error (rms) of 0.27 to 0.5, respectively using the data of the stations having good signal to noise ratio. The fault plane solutions (FPS) of these events have been determined using 12 local stations waveform data (NPL, AYAN, JHJR, KUDL, SONA, UJWA, GNR, JMIU, KKR, NRLA, NDI and BISR) (Fig. 2). The ISOLA software package has been used to determine the FPS. A correlation coefficient (between the observed and synthetic data) of > 0.5 and the double couple percentage (DC %) of > 50% in the moment tensor decomposition have been considered for finalizing the FPS (For details see supplementary data). Addionally, we also determined the Fault Plane Solutions of the events using first motion data (supplementary Figs. 6 and 7). In this exercise, FOCMAC subroutine in SEISAN software package has been used. In all cases FPS obtained by first motion polarity are consistent (in terms of fault mechanism) with the FPS obtained by waveform inversion technique. However, strike, dip and rake values are found to be different but agreed closely with the data derived by waveform inversion. The strain energy has been estimated for Delhi region using the standard formulation suggested by Kanamori^54^ to examine its spatial distribution. The earthquake catalog of the Delhi region for the period of 1998–2020 was used for the purpose (For details see supplementary data). The FPS computed in the present study have been compared with the earlier FPS in Delhi region to help in refining the current understanding of subsurface structural trends. The past works on geophysical, geological and seismological studies have been reviewed and a link between trends suggested by the FPS, energy estimation and the subsurface structures has been established.

## Supplementary Information

Supplementary Information.
