# Supplementary material for: A holistic seismotectonic model of Delhi region
Source: Sci Rep. 2021 Jul 5;11:13818. doi: 10.1038/s41598-021-93291-9 (PMC8257579; doi:10.1038/s41598-021-93291-9)
Supplement: Supplementary file 1 — Supplementary Information. [file 41598_2021_93291_MOESM1_ESM.docx]

**A Holistic Seismotectonic Model of Delhi Region**

Brijesh. K. Bansal^1,2^, Kapil Mohan^1*^, Mithila Verma^2^ and Anup K.Sutar^3^

^1^National Center for Seismology, Ministry of Earth Sciences, Lodhi Road, New Delhi-110003

^2^Geoscience/Seismology Division, Ministry of Earth Sciences, Lodhi Road, New Delhi-110003

^3^Borehole Geophysics Research Laboratory, Ministry of Earth Sciences, Karad, Maharashtra India

Corresponding author: Kapil Mohan

Email: kapil_geo@yahoo.co.in

**Earthquakes of Delhi Region**

A total of 122 earthquakes of magnitude M ≥ 3.0 occurred in Delhi region from January-2001 to 10^th^ June 2020 (**Supplementary Fig. 1a**). The depth distribution shows that the focal depths generally lie within 15 km from the surface with only about 10% of events deeper than 15km (**Supplementary Fig. 1b**). The maximum number of earthquakes with magnitude M ≥ 3.0 in a particular year were recorded in 2012 (N=15). The quietest year was 2009 with one event only.


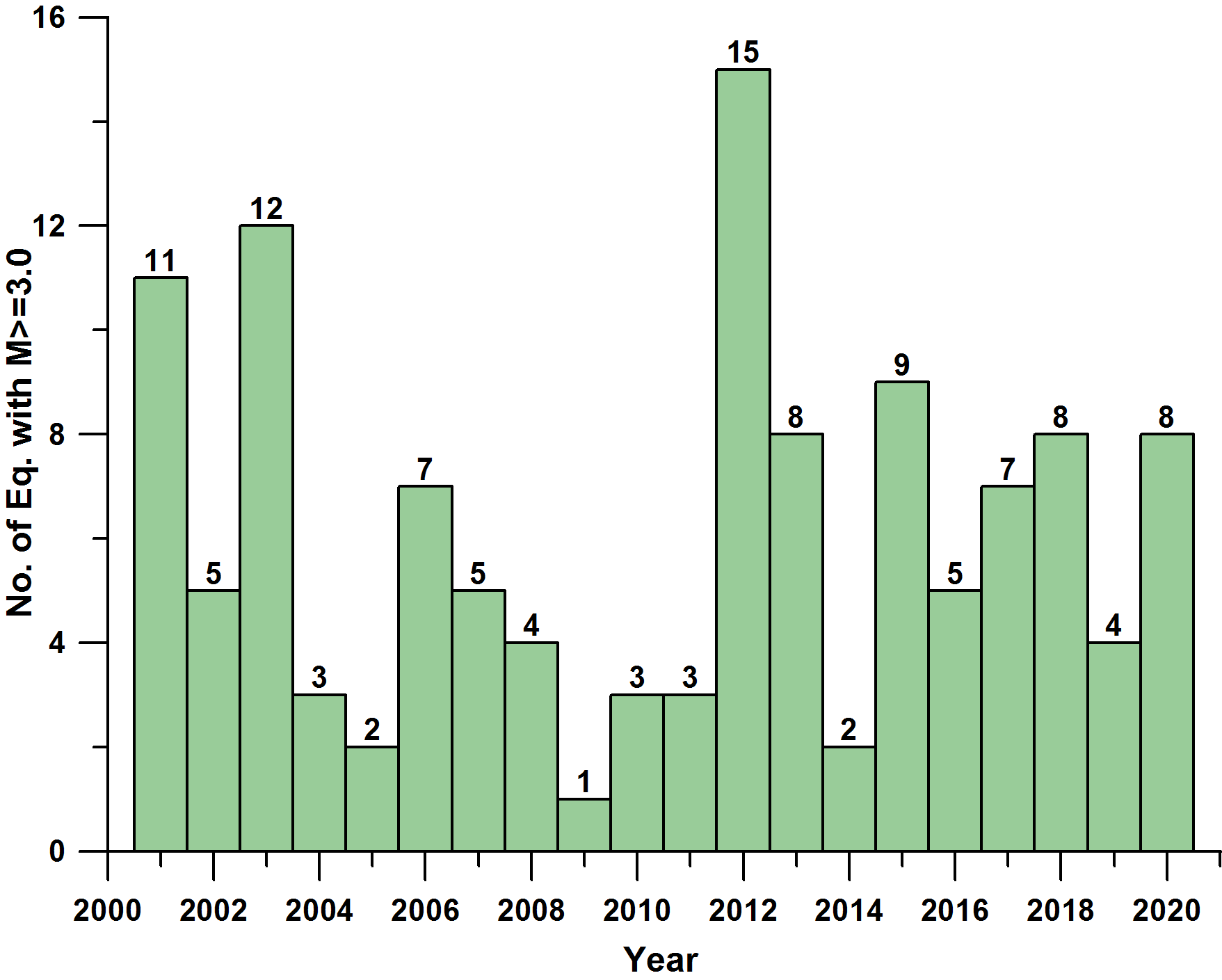


**(a)**


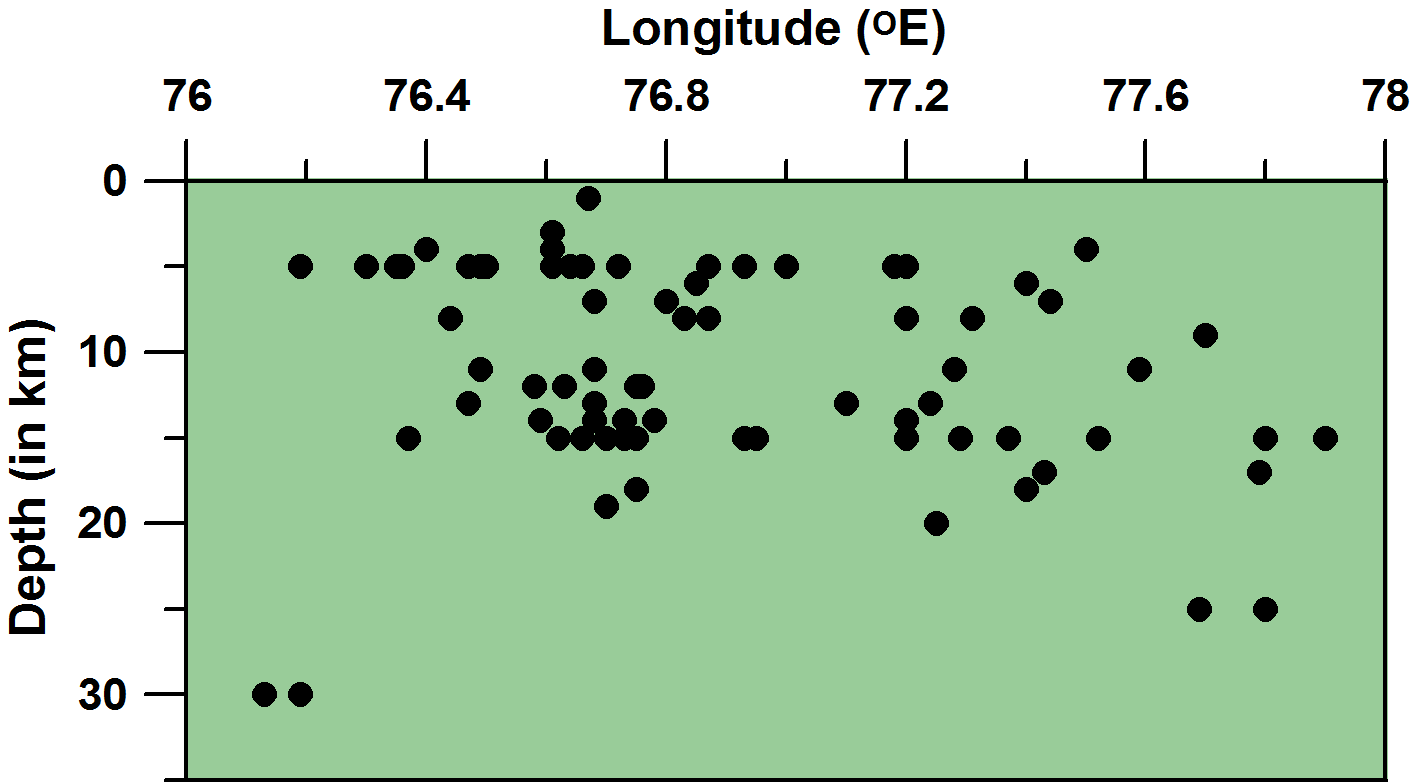


**(b)**

**SupplementaryFigure 1:** (a) Plot showing the number of earthquakes with magnitude M≥3.0 that occurred in the Delhi region from 01.01.2001 to 10.06.2020; (b) Depth distribution of the earthquakes with magnitude M≥3.0.

**Fault plane solutions**

In the present analysis, we have used full wave form inversion and P-wave first motion polarity methods to find fault plane solutions of smaller magnitude events occurred in Delhi region.

Details on methodology is given below:

**P-Wave first motion:**

In this method, a small sphere is assumed surrounding the earthquake source, called as focal sphere. Any P-wave ray path leaving the source is identified by the azimuth from the source ($\phi_{s}$) and the ray parameter or take off angle (*i*). Each ($\phi_{s})$ and (*i*) combination describes a unique path through the source to a point on the focal surface. The corresponding portion of the outgoing wave front conveying the initial motion in the associated region of the outgoing wave. Stereographic or equal-area projections are used to project the focal sphere onto a plane and the take-off angle (i) and azimuth ($\phi_{s}$) of the ray can be plotted on the same plane. Combining the first P-wave polarity of the observed ray path from a number of stations, the fault plane associated with the event can be inferred using this approach ([Stein](https://www.wiley.com/en-us/search?pq=%7Crelevance%7Cauthor%3ASeth+Stein) and [Wysession](https://www.wiley.com/en-us/search?pq=%7Crelevance%7Cauthor%3AMichael+Wysession)^1^). In this study, we used FOCMAC program to obtain the fault plane solution, which is included in the SEISAN software package^2^ . The program makes a grid-search and obtain how many polarities and amplitude ratios are best fit for each possible fault plane solution. The obtained FPS using P-wave first motion data provides a first-hand information on the type of faulting as well as strike and dip information for the causative source due to unavailability of well azimuthally distributed stations surrounding the source^3^. The final solution for the events, however, is accepted for the FPS obtained using full waveform inversion.

**Full Waveform inversion**:

The source mechanisms of a seismic event can be represented by a symmetric matrix (M) represented as,

|  | $M=\left( \begin{matrix} M_{11} & M_{12} & M_{13} \\ M_{21} & M_{22} & M_{23} \\ M_{31} & M_{32} & M_{33} \end{matrix} \right)$, | (1) |
| --- | --- | --- |

where each element of the matrix represents a force couple and M is termed as full moment tensor matrix. The minimum (*λ*_min_) and maximum (*λ*_max_) eigenvalues of moment tensor matrix (M) corresponds to maximum tensile (T) and compressional stress (P) directions.

The scalar seismic moment is defined as the Euclidian norm of the M^4^ .

|  | $M_{0}=\sqrt{\frac{\sum_{p=1}^{3} \sum_{q=1}^{3} (M_{pq})^{2}}{2}}$ | (2) |
| --- | --- | --- |

Further, the displacement ‘u’ by a point source with a given position that give rise to seismic waves can be expressed by means of moment tensor ***M*** and spatial derivative of Green’s tensor ***G***^5^:

|  | $u_{i}(t)=\sum_{p=1}^{3} \sum_{q=1}^{3} M_{pq}*{G_{ip,q}^{'}}$ | (3) |
| --- | --- | --- |

where * stands for temporal convolution, and *p, q* denotes three Cartesian coordinates. The moment tensor can be expressed in the form of a linear combination of six elementary (dimensionless) tensors **M^i^ .**

|  | $M_{pq}=\sum_{i=1}^{6} a_{i}M_{pq}^{i}$. | (4) |
| --- | --- | --- |

This is a convenient parametrization of the source and is characterized by six scalar coefficients a_i_^6,7^ .

Combining (3) and (4) yields:

|  | $u_{i}(t)=\sum_{p} \sum_{q} (\sum_{j=1}^{6} a_{j}M_{pq}^{j})*G_{ip,q}$ | (5) |
| --- | --- | --- |

In matrix notation

|  | **u = E a** . | (6) |
| --- | --- | --- |

The (formally) over-determined linear inverse problem (6) for **a** can be solved by the least-squares method

|  | **a_opt_** = **(E^T^E)^-1^ E^T^u** | (7) |
| --- | --- | --- |

This least-squares formulation is followed from Kikuchi and Kanamori^8^

The grid search maximizes the correlation between the observed (**u**) and synthetic (**s**) seismograms given by:

|  | $Corr=\frac{\int us}{\sqrt{\int u^{2}s^{2}}}$, | (8) |
| --- | --- | --- |

where $\int us=\sum_{i} \int u_{i}(t)s_{i}(t)dt$ and summation is over components and stations.

The traditional decomposition **M** = **M**_ISO_+ **M**_DEV_, where **M**_ISO_ and **M**_DEV_ are the isotropic and deviatoric parts, respectively. Furthermore, **M**_DEVIA_ = **M**_DC_ + **M**_CLVD_ (e.g., Julian ^9^), where **M**_DC_ is the de double couple and **M**_CLVD_ is the Compensated Linear Vector Dipole part. The decomposition provides percentage of source components.

We have used the ISOLA software package ^10^ for estimation of fault plane solution. This code has been applied by many researchers across the globe^,11,12,13,14^.

We have used the data from more than 22 seismic stations are used to locate the earthquakes of April 12 (M3.5), May 10 (M3.4), May 29, 2020 (M4.4) and June 01, 2017 (Mw4.2). However, the May 29, 2011 (Mw3.4) event was recorded by 8 stations. The events have location errors of < 1.5 km in latitude/longitude and <2.7 km in depth. The fault plane solutions (FPS) of the events are obtained using only selected numbers of stations with the ISOLA software package^10^.

Well recorded seismic data within 100 km radius from the epicentre of the events are used in the waveform inversion. The waveforms with cut off SNR>2 in the frequency range of interest are used in the inversion. Various velocity models of the study region (such as, Chun^15^; Suresh et al.^16^; Kumar et al.^17^; Mitra et al.^18^) are tested and the model of Mitra et al.^18^ is found to be the most suitable to compute synthetic waveform with high correlation coefficient and double couple percentage (DC%).

Some seismic stations as well as components of the simulated waveforms (Supplementary Figs. 2a, 3a, 4a and 5a) that showed negative correlation and did not match with observed waveforms are excluded from the inversion to get FPS. A vertical grid search at various trial depths was performed to get the best solution for all the events (Supplementary Figs. 2b, 3b, 4b and 5b). The best solution was selected after getting global correlation coefficient >0.5 and DC% > 50.

In addition, we have also incorporated first motion data to get Fault Plane Solutions of May 10, 2020 (M3.4) event. In this exercise, we have made use of FOCMAC subroutine in SEISAN software package. P-wave first motion polarities are picked and then searched for the best solution using the FOCMAC subroutine. The result shows a normal with strike slip movement (Supplementary Fig. 6). The obtained solution shows a strike of 197^o^, dip of 49^o^ and rake of -78^o^. Similarly, FPS of May 29, 2020 (M4.5) event is obtained by first motion polarity data (Supplementary Fig. 7). In all cases FPS obtained by first motion polarity are consistent (in terms of fault mechanism) with the FPS obtained by waveform inversion technique. However, strike, dip and rake values are found to be different but agreed closely with the data derived by waveform inversion.


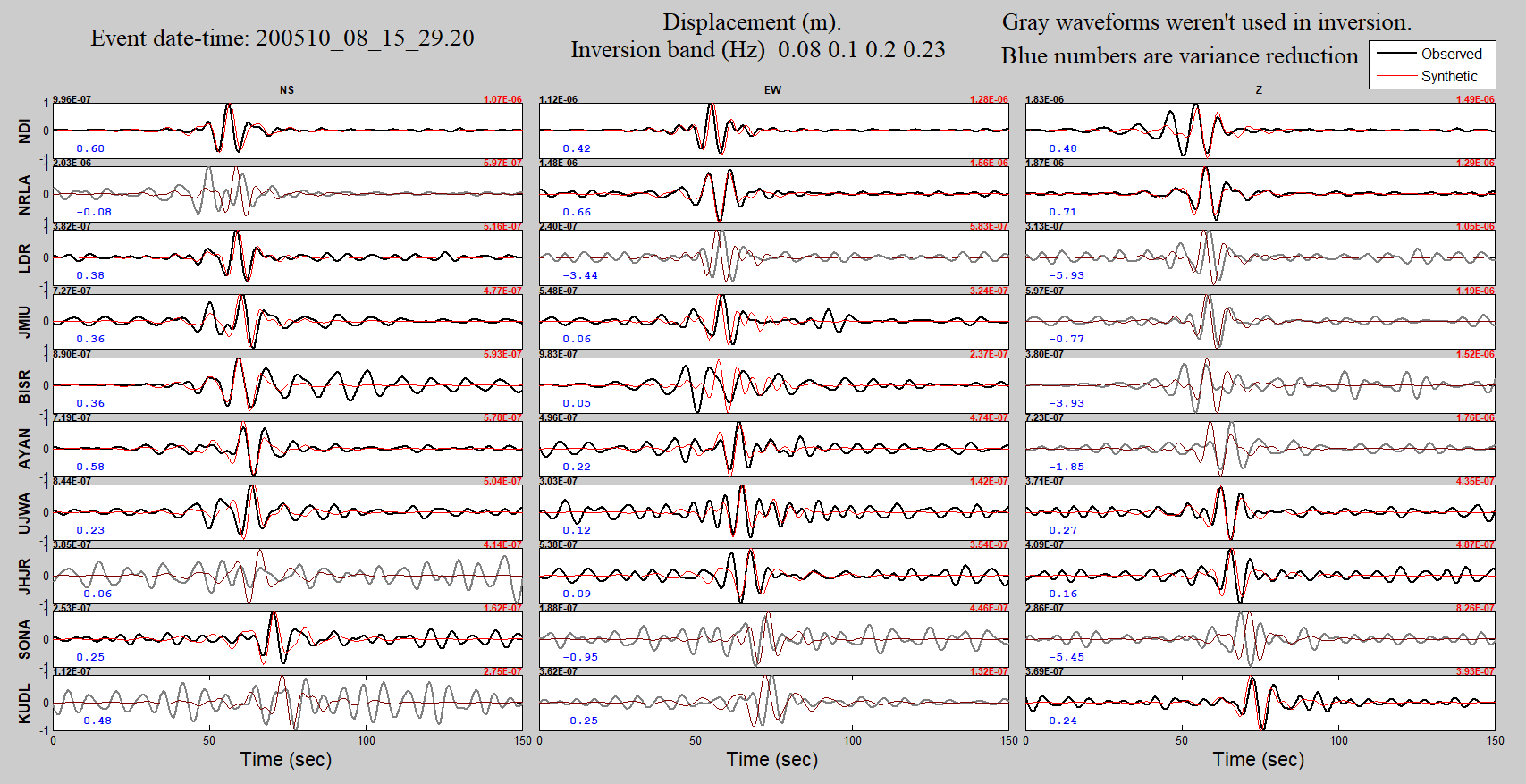


**Supplementary figure 2a:** Normalized correlation plot between observed and synthetic displacement waveforms of May 10, 2020 event (M 3.4), in three components at 10 different seismic stations. Waveforms data have been band-pass filtered in the frequency range (0.1- 0.2) Hz. Black and red lines represent observed and synthetic seismograms respectively and values (in blue color) on the lower left corner of each component represent the correlation coefficient. Grey waveforms are excluded from the inversion.


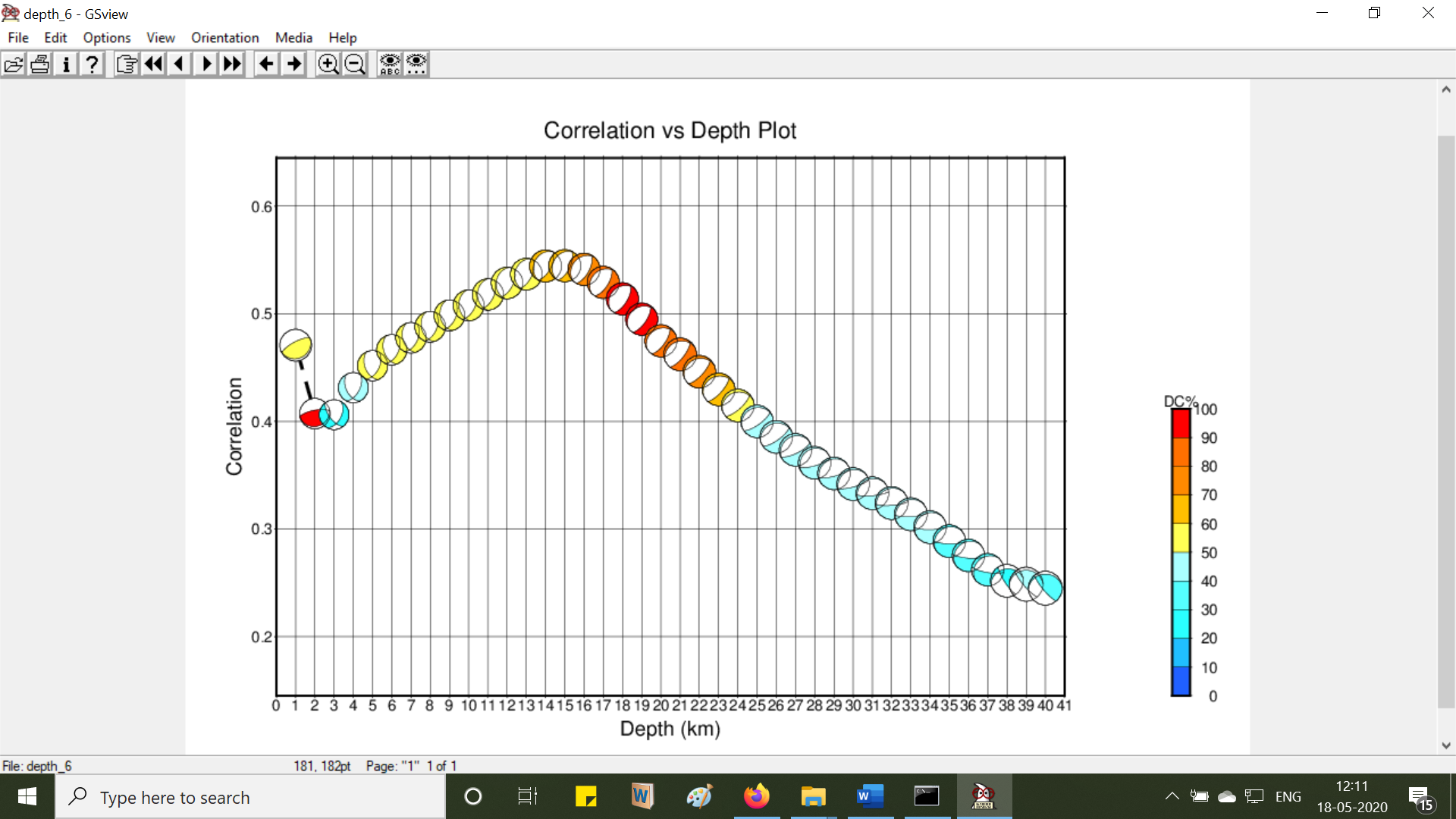


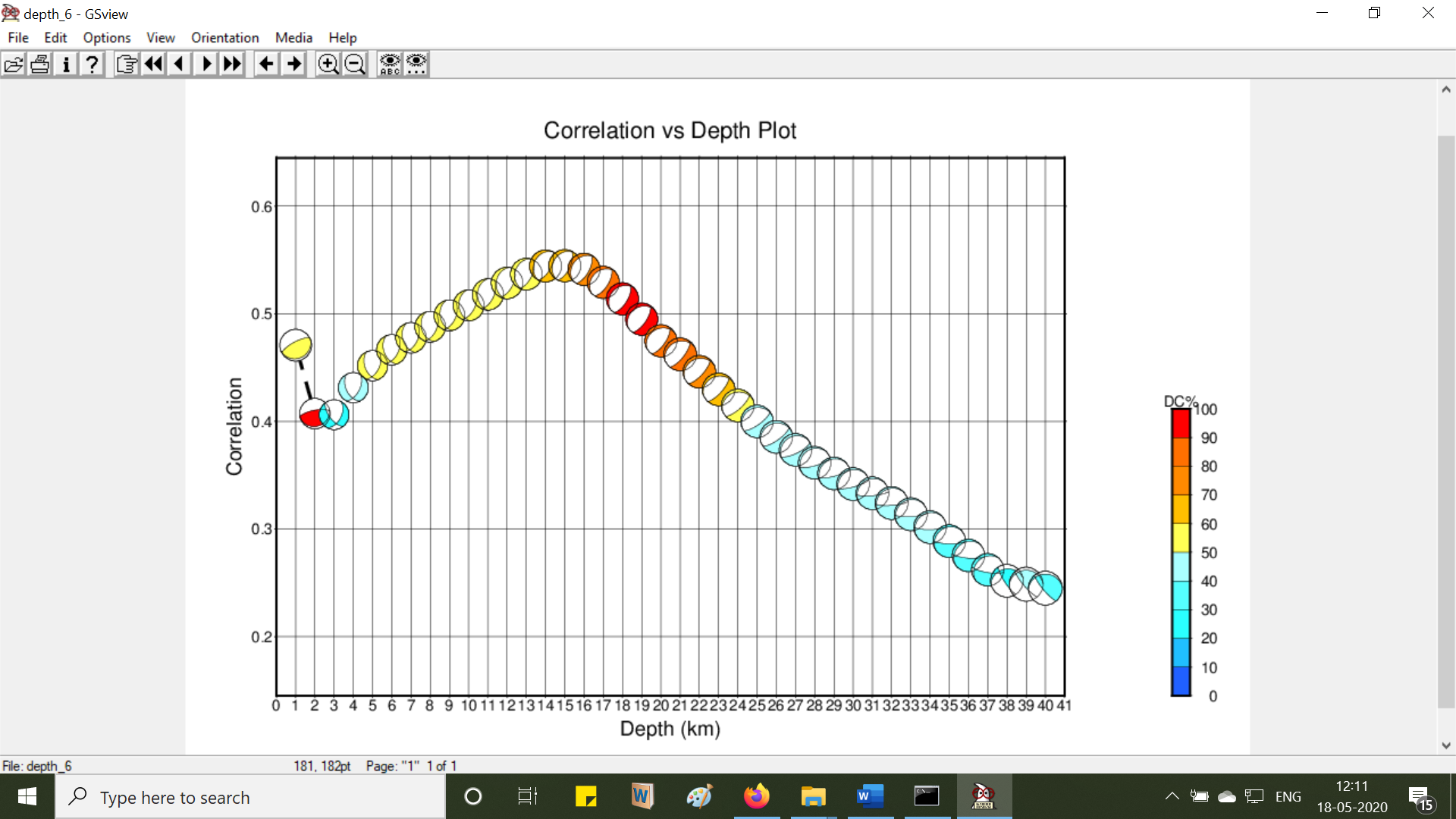


**Supplementary figure 2b:** Variation of correlation and DC% at different depth for the 10^th^ May 2020 (M 3.4) event to find the best source depth. The DC% has been scaled and given on the right side of the figure.


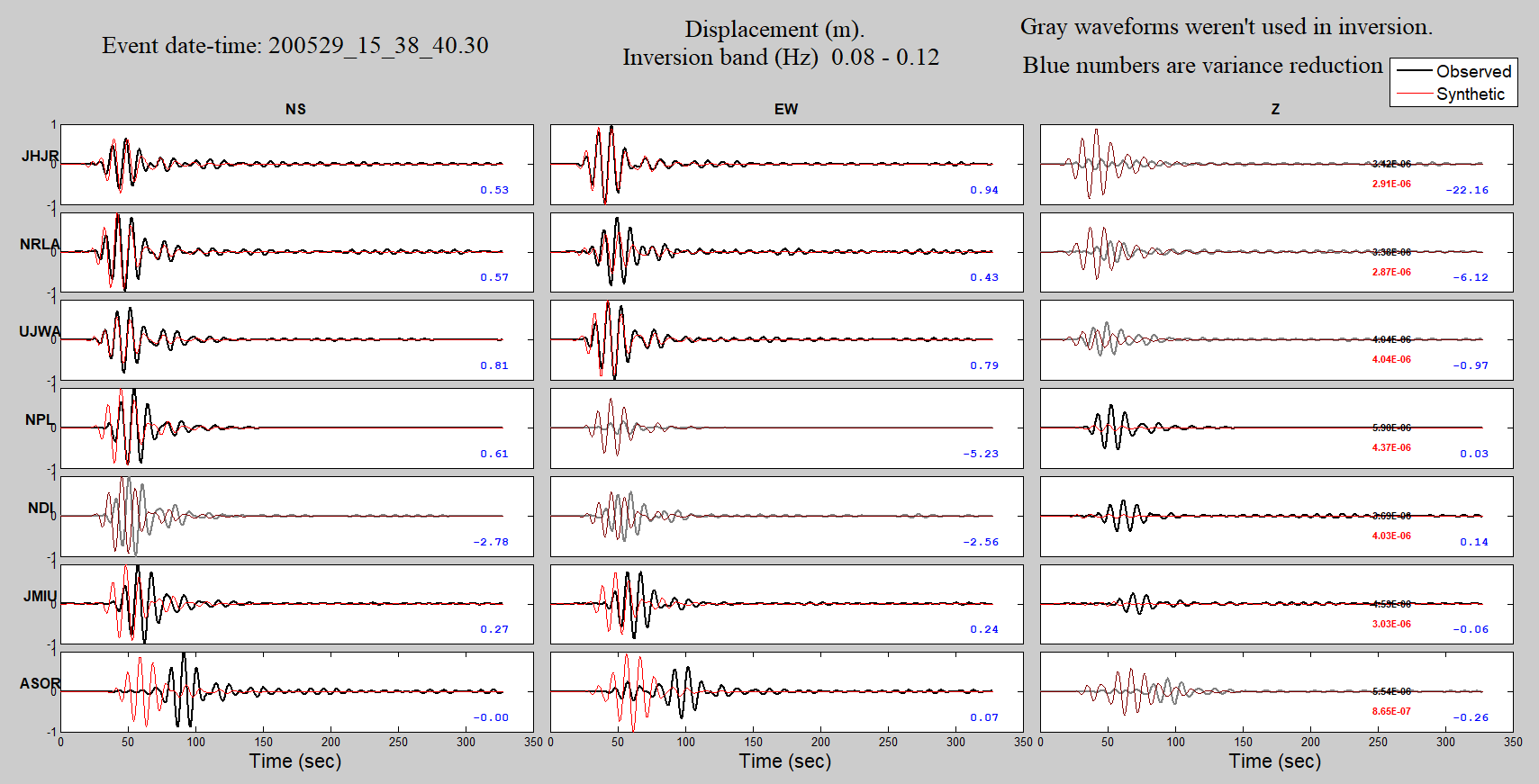


**Supplementary figure 3a:** Normalized correlation plot between observed and synthetic displacement waveforms of May 29, 2020 event (M 4.4), in three components at 7 different seismic stations. Waveforms data have been band-pass filtered in the frequency range (0.08- 0.12) Hz. Black and red lines represent observed and synthetic seismograms respectively and values (in blue color) on the lower right corner of each component represent the correlation coefficient. Grey waveforms are excluded from the inversion.


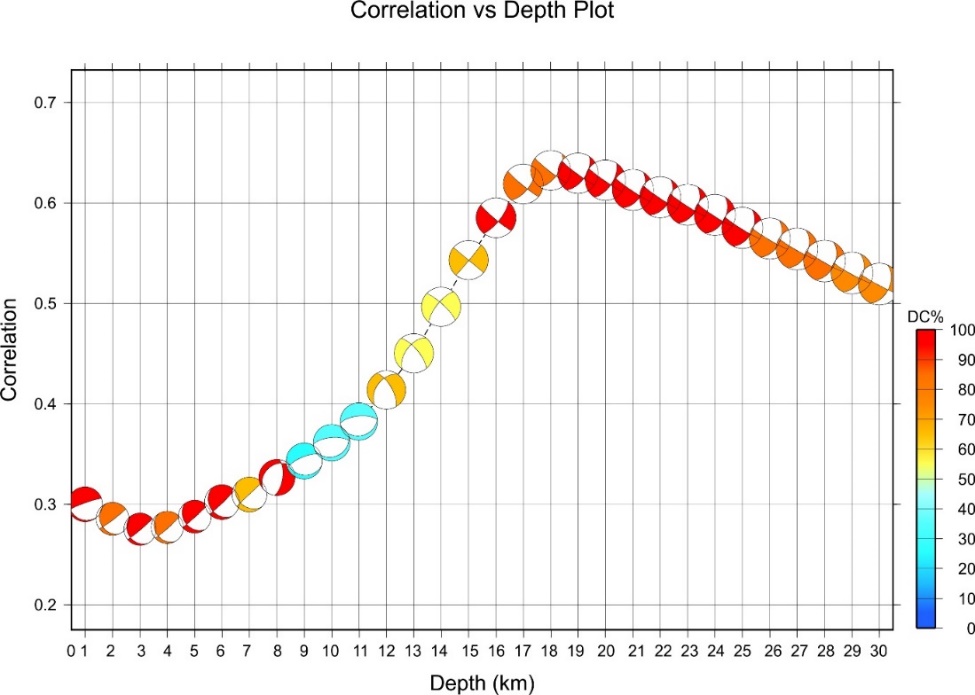


**Supplementary figure 3b:** Variation of correlation and DC% at different depth for the 29^th^ May 2020 (M 4.4) event to find the best source depth. The DC% has been scaled and given on the right side of the figure.


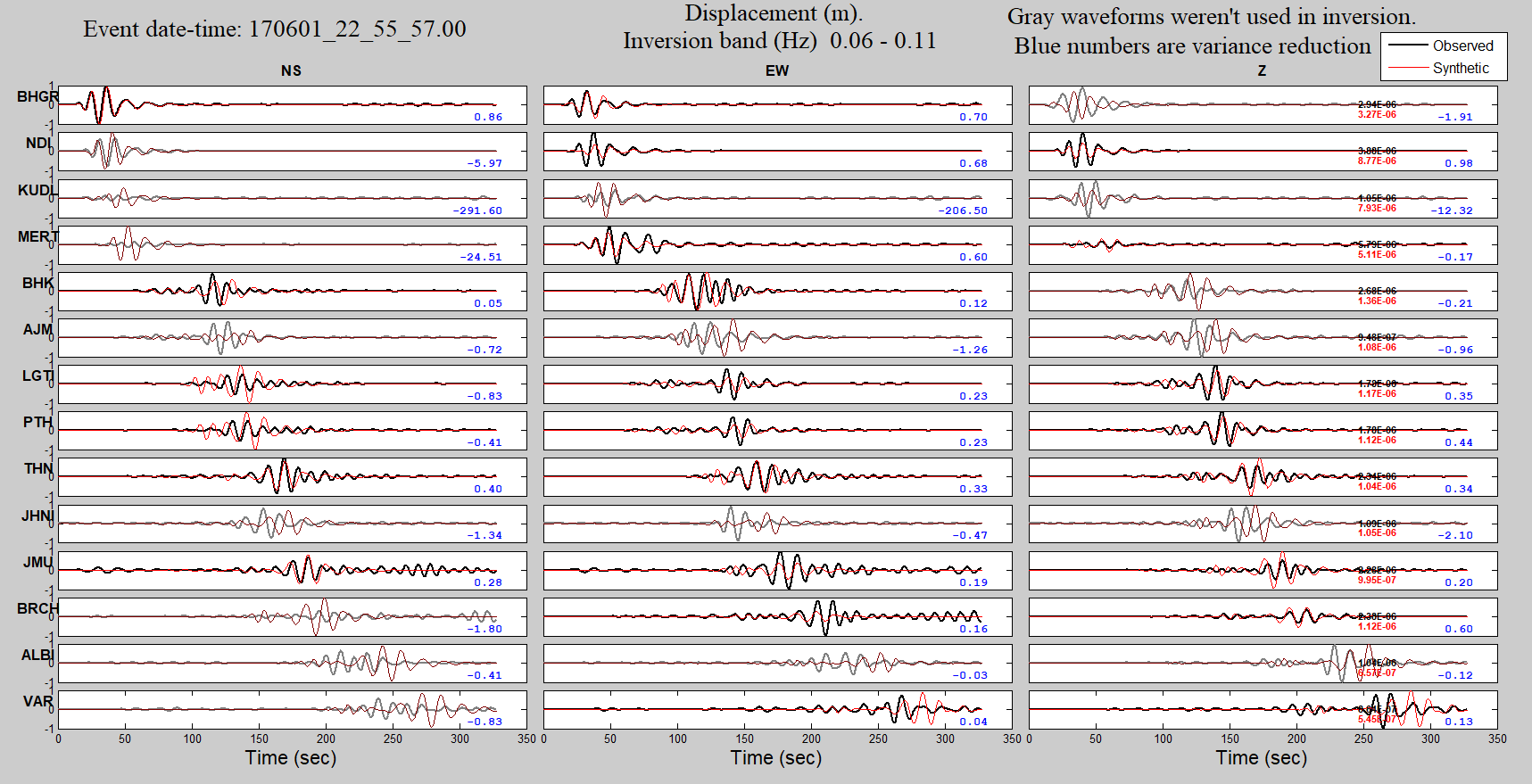


**Supplementary figure 4a:** Normalized correlation plot between observed and synthetic displacement waveforms of June 01, 2017 event (M 4.2), in three components at 14 different seismic stations. Waveforms data have been band-pass filtered in the frequency range (0.06- 0.11) Hz. Black and red lines represent observed and synthetic seismograms respectively and values (in blue color) on the lower right corner of each component represent the correlation coefficient. Grey waveforms are excluded from the inversion.


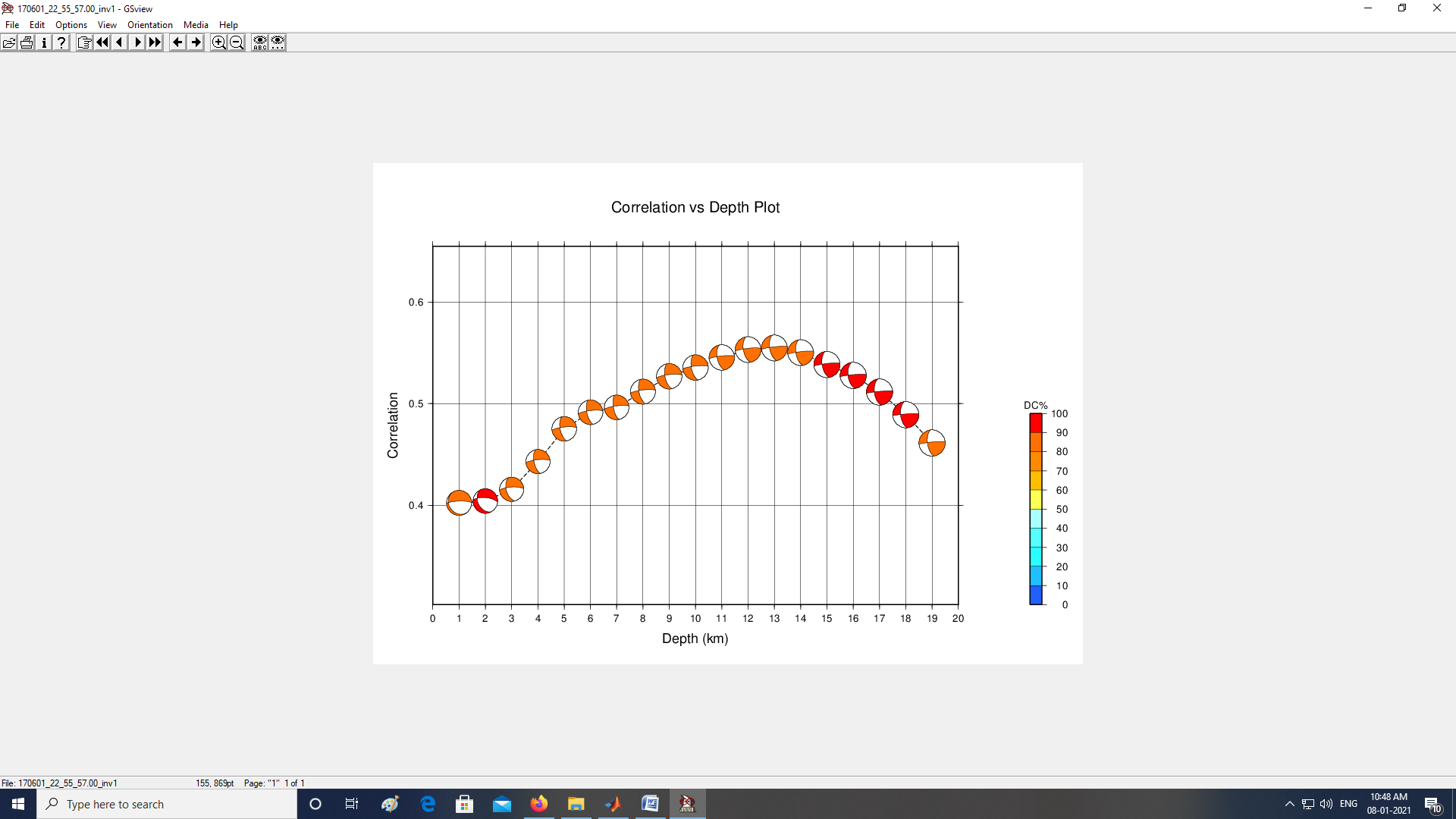


**Supplementary figure 4b:** Variation of correlation and DC% at different depth for the June 01, 2017 event (M 4.2) event to find the best source depth. The DC% has been scaled and given on the right side of the figure.


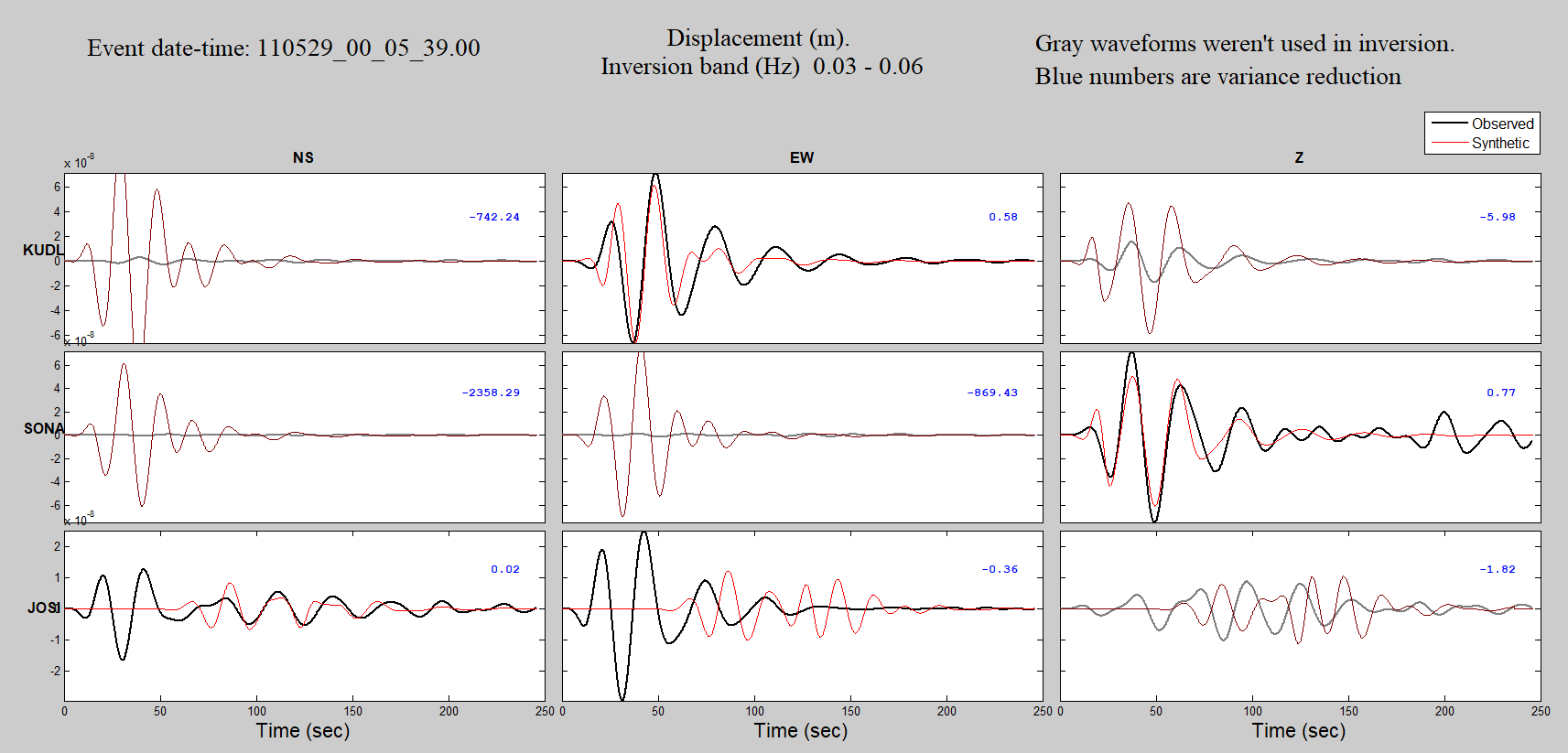


**Supplementary figure 5a:** Normalized correlation plot between observed and synthetic displacement waveforms of May 29, 2011 event (M 3.4), in three components at 3 different seismic stations. Waveforms data have been band-pass filtered in the frequency range (0.03- 0.06) Hz. Black and red lines represent observed and synthetic seismograms respectively and values (in blue color) on the upper right corner of each component represent the correlation coefficient. Grey waveforms are excluded from the inversion. Grey waveforms are excluded from the inversion


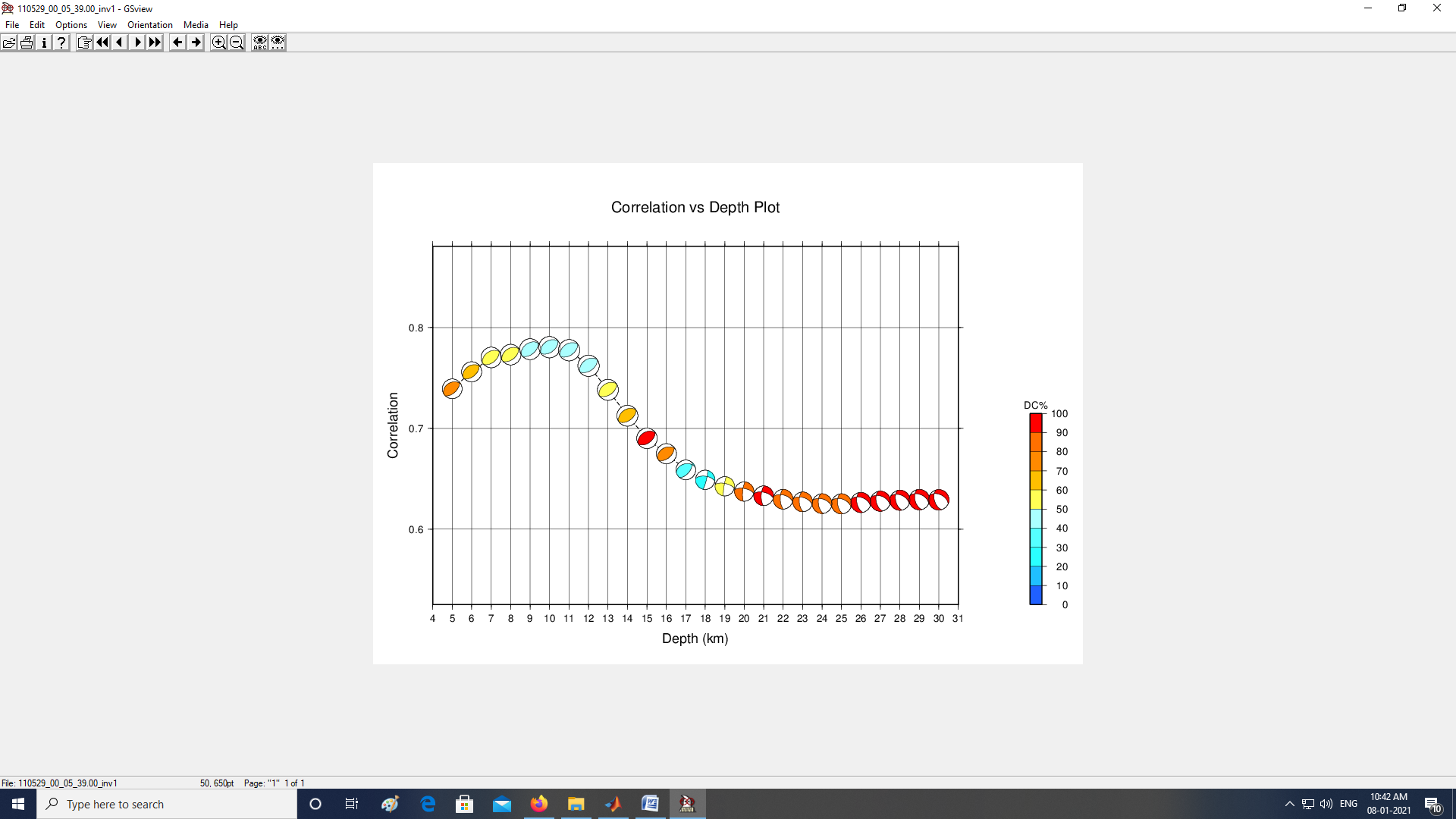


**Supplementary figure 5b:** Variation of correlation and DC% at different depths for the May 29, 2011 event (M 3.4) event to find the best source depth. The DC% has been scaled and given on the right side of the figure.


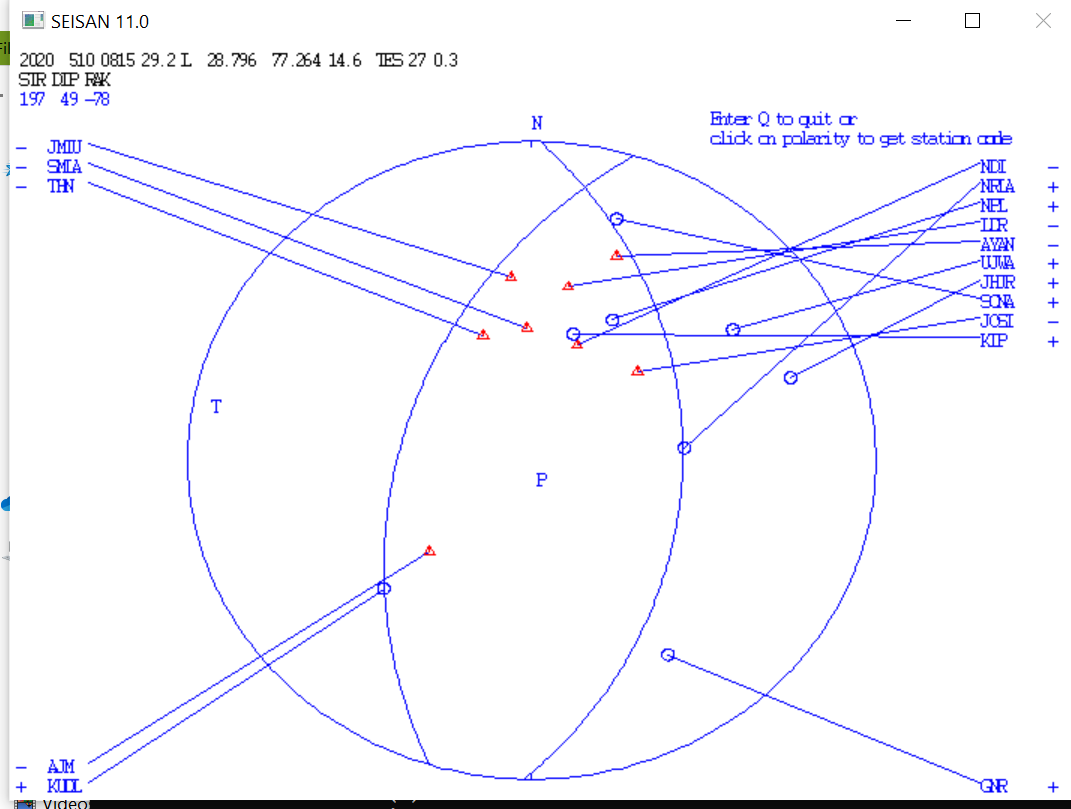


**Supplementary figure 6:** FPS of May 10, 2020 (M3.4) event obtained by P-wave first motion polarity data.


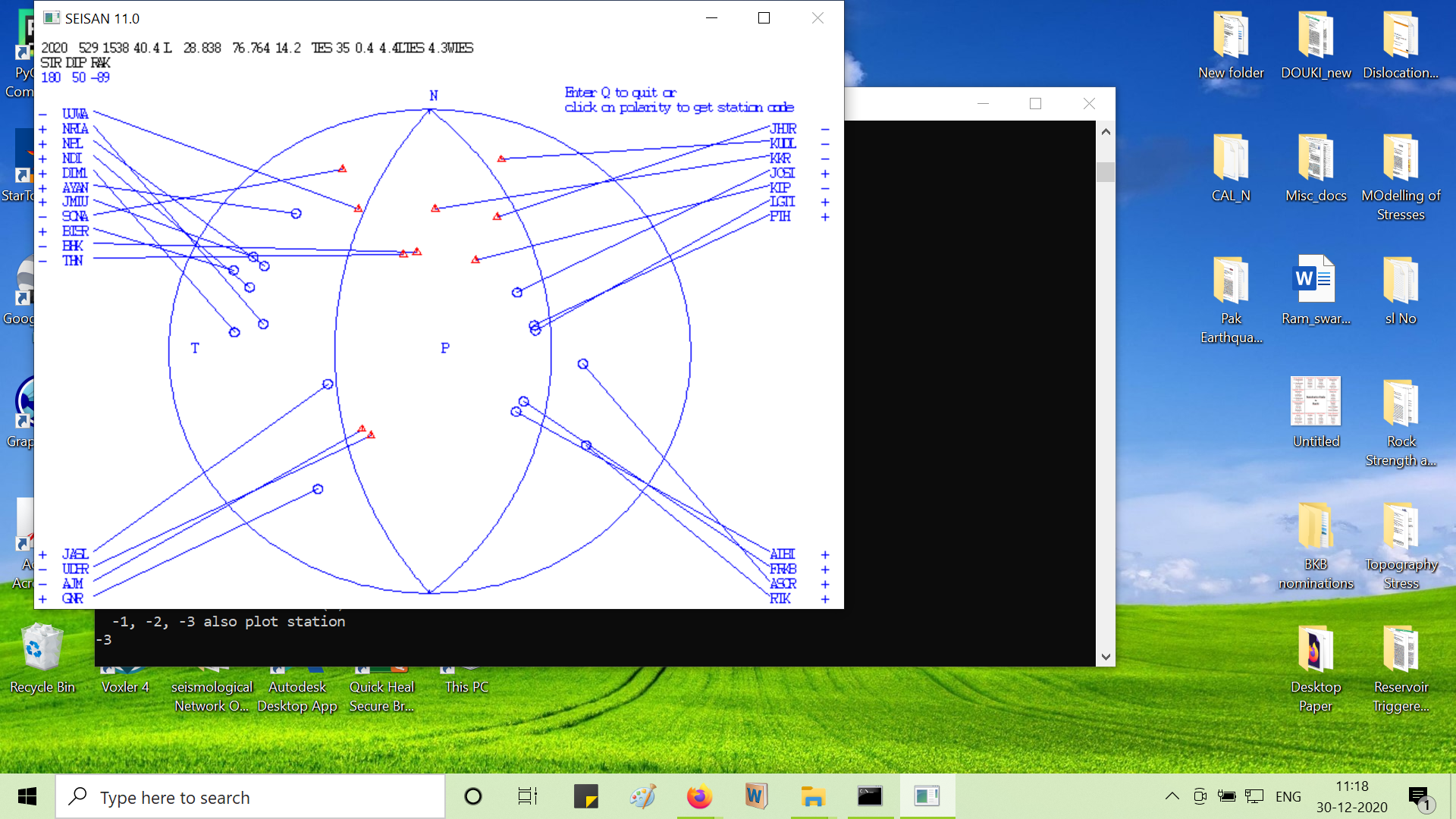


**Supplementary figure 7:** FPS of May 29, 2020 (M4.5) event obtained by P-wave first motion polarity data.

**Distribution of strain energy in the Delhi region**

The spatial distribution of strain energy has been estimated for Delhi and the surrounding areas based on the earthquake catalog of the region for the period 1998-2020 taken from the website of National Centre for Seismology (<https://seismo.gov.in/content/seismological-data>). Earthquakes with magnitudes ranging between M0.8 and M5.1 have been considered. The potential energy $J_{p}$ of a volume of rock (W) strained by an amount S immediately after the earthquake is given by^19^:

$$J_{p}=0.5\mu WS^{2}$$

where $\mu$ is the shear modulus of the rock volume. The energy released by the seismic waves can be expressed as:

$J_{p}=0.5 \mu WS^{2}f$,

where *f* is the fraction of energy released as seismic waves. The energy released by an earthquake is related to the magnitude of the earthquake and can be written as (after Guttenberg and Richter^20^):

$$\log J=11.8+1.5 M$$

where, M is the magnitude of the earthquake. The strain released in the process of an earthquake can be estimated from the energy released using the formulation given by Benioff^19^ and is proportional to the square root of energy ($\sqrt{J}$).

Although different magnitude scales were used for the estimation of earthquake energy, the moment magnitude scale is preferred over other magnitude scales as it is based on the total moment release of the earthquake. Earthquake moment is a product of the distance a fault moved and the force required to move it and works over a wide range of earthquake sizes^21^.

Later, [Kanamori](http://www.gps.caltech.edu/faculty/kanamori/kanamori.html)^22^ came up with a relationship between seismic moment and seismic wave energy, which is given by:

$$J=\frac{M_{o}}{20000}$$

where M_o_ is the seismic moment. In the present study, we have used the Kanamori^22^ relationship to estimate the energy released during the event and to estimate seismic moment (M_o)._ We have used different regression relations between M_L_ and Mw of the stable continental region^23,24,25^ and have taken the average estimate of the energy as the final output. The average error associated while converting M_L_ to M_w_ is found to be 0.3 with a standard deviation of 0.07. For the spatial variation of energy, the region is divided into 0.3^o^ x 0.3^o^ grid and the events collected within the grids are used to estimate the energy in Joules (J). The average energy is estimated for the collected events and the mean value is assigned at the center of the grid. A contour map is prepared to show the spatial variation of the energy released and given as **Fig.4**.

**Supplementary Table 1:** Source parameters of earthquakes computed in the previous studies of Delhi region.

| **Origin date and time** | **Lat**  **(^o^N)** | **Long(^o^E)** | **Mag.** | **Focal depth, km** | **Str1** | **Dip1** | **Rak1** | **Str2** | **Dip2** | **Rak2** |
| --- | --- | --- | --- | --- | --- | --- | --- | --- | --- | --- |

05/03/2012^*^ 28.748 76.603 4.9 (ML) 9.0 347.9◦ 47.85◦ 131.34◦ 115.26◦ 56.18◦ 53.88◦
13:11:04.7 5.0 (Mw)
(IST)

08/04/2001^#^ 28.629 77.153 3.4 14.6 65◦ 60◦ -21◦ 03:06:28.0IST)

18/03/2004^#^ 28.627 77.226 2.6 7.9 45◦ 85◦ -110◦ 07:52:33.2

(IST)

25/11/07** 28.57 77.10 4.1(Mw) 30.0 31◦ 86◦ -35◦ 124◦ 55◦ 175◦

11:12:00

(UTC)

*26,^#^27,^**^28

**Supplementary Table 2:** List of significant earthquakes in and around Delhi since 1720 A.D. Distance from Delhi is measured from India Meteorological Department, Lodhi Road, New Delhi

**Date Lat Long M Region Distance from Reference**

**(◦N) (◦E) Delhi, km**

16/07/ 1720 28.37 77.10 6.5 * Delhi 27 IMD

28.70 77.20 7.4 Delhi GSHAP

Catalogue

16/01/1842 27.00 78.00 5.0* Near Mathura 192 IMD

10/10/1956 28.15 77.67 6.7 Near Bulandshahar 65 IMD

27/08/1960 28.20 77.40 6.0 Near Faridabad 46 IMD

28.48 77.0 4.8 Between Delhi Singh et al.^29^

(M_W_) Cantonment and

Gurugram

15/08/1966 28.67 78.93 5.8 Near Moradabad 167 IMD/NCS

*estimated from macroseismic data^30^.

**References:**

1. Stein, S. and Wysession, M. An Introduction to Seismology, Earthquakes, and Earth Structure. e-Book, Wiley-Blackwell, ISBN: 978-1-118-68745-1(2013).

2.Ottemöller, L., Voss, P. & Havskov, J. Seisan earthquake analysis software for Windows, Solaris, Linux and MacOSX (2017).

3.Lentas, K. Towards routine determination of focal mechanisms obtained from first motion P-wave arrivals. Geophys J Int, **212**(3), 1665-1686 (2018).

4.Silver, P.G. and Jordan, T. H. Optimal estimation of scalar seismic moment, Geophys J Int, **70** (3), 755–787 (1982).

5.Aki, K., Richards, P.G. Quantitative seismology. University Science Books, 2nd ed, Sausalito, CA (2002).

6.Bouchon, M. A simple method to calculate Green's functions for elastic layered media. Bull Seismol Soc Am **71**(4): 959-971(1981).

7. Coutant, O. Numerical study of the diffraction of elastic waves by fluid-filled cracks. J Geophys Res **94:**17805 (1989).

8. Kikuchi, M. and Kanamori, H. Inversion of complex body waves—III. Bull Seismol Soc Am **81** (6): 2335–2350 (1991).

9. Julian, B. R., Miller, A. D. and Foulger, G. R. Non-double-couple Earthquakes. Rev. Geophys **36**, 525-549(1998).

10. Sokos, E., Zahradník J. Evaluating Centroid-Moment-Tensor Uncertainty in the New 29 Version of ISOLA Software (2013).

11. Baruah, S. and Boruah, M. Waveform Modelling of 2009 Bhutan Earthquake of Magnitude 6.1 (Mw) Using Local Network Data of North East India. In: D'Amico S. (eds) Moment Tensor Solutions. Springer Natural Hazards. Springer, Cham. <https://doi.org/10.1007/978-3-319-77359-9_18> (2018).

12. Verma M., Sutar, A.K., Bansal, B.K., Arora, B.R., Bhat, G.M. MW 4.9 earthquake of 21 August, 2014 in Kangra region, Northwest Himalaya: Seismotectonics implications,
J Asian Earth Sci, **109**, 29-37 (2015).

13. Parija M.P., Kumar, S., Tiwari, V.M., Purnachandra Rao, N., Kumar, N., Biswal, S, Singh, I. Microseismicity, tectonics and seismic potential in the Western Himalayan segment, NW Himalaya, India, J Asian Earth Sci, 159, 1-16(2018).

14. Liu, J., & Zahradník, J. The 2019 MW 5.7 Changning earthquake, Sichuan Basin, China: A shallow doublet with different faulting styles. Geophys. Res. Lett., **47**(4), e2019GL085408. (2020).

15. ChunK-Y. Crustal block of the western Ganga basin: a fragment of oceanic affinity? Bull Seismol Soc Am **76** (6), 1687–1698 (1986).

16. Suresh, G., Jain, S. & Bhattacharya, S.N.  Lithosphere of Indus block in the north western Indian subcontinent through Genetic Algorithm inversion of surface-wave dispersion. Bull Seismol Soc Am. **98** (4), 1750–1755 (2008).

17. Kumar, N., Sharma, J., Arora, B.R. & Mukhopadhyay, S. Seismotectonic model of the Kangra–Chamba sector of Northwest Himalaya: constraints from joint hypocentre determination and focal mechanism. Bull Seismol Soc Am **99**, 95–109 (2009).

18. Mitra, S., Kainkaryam, S.M., Padhi, A., Rai, S.S., &Bhattacharya, S.N. The Himalayan foreland basin crust and upper mantle. Phys Earth Planet Inter. **184** (1–2), 34–40 (2011).

19.Benioff, H. Seismic evidence for the fault origin of ocean deep. *Bull. Geol. Soc. Am.***60**, 1837-1848 (1949).

20.[Gutenberg](javascript:;), B. & [Richter](javascript:;), C.F. Earthquake magnitude, intensity, energy, and acceleration: (Second paper), *Seismol. Soc. Am., Bull.* **46** (2), 105–145 (1956).

21.Vassiliou, M. S. & Kanamori, H. The energy release in earthquakes. *Bull. Seismol. Soc. Am.*, **72** (2), 371–387 (1982).

22.Kanamori, H. Magnitude scale and quantification of earthquakes. *Tectonophysics* **93**, 185-199 (1983).

23.Johnston, A.C. Seismic moment assessment of earthquakes in stable continental regions-I. Instrumental seismicity, *Geophys. J. Int.* **124**, 381–414 (1996).

24.Schulte, S.M. & Mooney, W.D. An updated global earthquake catalog for stable continental regions—Reassessing the correlation with ancient rifts. *Geophys. J. Int.* **161**, 707–721(2005).

25.Kolathayar, S. & Sitharam, T. G. Characterization of Regional Seismic Source Zones in and around India. *Seismol. Res. Lett.* 83 (1), 77–85 (2012).

26.Bansal, B.K. & Verma, M. The M 4.9 Delhi earthquake of 5 March 2012, *Curr. Sci.*, **102**(12), 1704-1708 (2012).

27.Bansal, B.K. et al. Source study of two small earthquakes of Delhi, India and estimation of ground motion from future moderate, local events. *Jour. Seismol*. **13**, 89-105(2009).

28.Singh, S.K. et al. Delhi earthquake of 25 November 2007 (Mw4.1): Implications for seismic hazard Curr. Sci. **99**(7), 939-947 (2010).

29.Singh, S. K. et al. The Delhi 1960 earthquake: epicentre, depth and magnitude *Curr. Sci.,***105**(8), 1155-1165 (2013).

30.Chouhan, R.K.S. Seismotectonics of Delhi Region. *Proc. Indian Nat. Sci. Acad.*, **41** (A), 429-447 (1975).
